# The rostromedial tegmental nucleus is essential for non-rapid eye movement sleep

**DOI:** 10.1371/journal.pbio.2002909

**Published:** 2018-04-13

**Authors:** Su-Rong Yang, Zhen-Zhen Hu, Yan-Jia Luo, Ya-Nan Zhao, Huan-Xin Sun, Dou Yin, Chen-Yao Wang, Yu-Dong Yan, Dian-Ru Wang, Xiang-Shan Yuan, Chen-Bo Ye, Wei Guo, Wei-Min Qu, Yoan Cherasse, Michael Lazarus, Yu-Qiang Ding, Zhi-Li Huang

**Affiliations:** 1 Department of Pharmacology, School of Basic Medical Sciences, State Key Laboratory of Medical Neurobiology, Institutes of Brain Science and Collaborative Innovation Center for Brain Science, Fudan University, Shanghai, China; 2 International Institute for Integrative Sleep Medicine (WPI-IIIS), University of Tsukuba, Tsukuba, Ibaraki, Japan; 3 Department of Anatomy and Neurobiology, School of Medicine, Tongji University, Shanghai, China; UCSF School of Medicine, United States of America

## Abstract

The rostromedial tegmental nucleus (RMTg), also called the GABAergic tail of the ventral tegmental area, projects to the midbrain dopaminergic system, dorsal raphe nucleus, locus coeruleus, and other regions. Whether the RMTg is involved in sleep–wake regulation is unknown. In the present study, pharmacogenetic activation of rat RMTg neurons promoted non-rapid eye movement (NREM) sleep with increased slow-wave activity (SWA). Conversely, rats after neurotoxic lesions of 8 or 16 days showed decreased NREM sleep with reduced SWA at lights on. The reduced SWA persisted at least 25 days after lesions. Similarly, pharmacological and pharmacogenetic inactivation of rat RMTg neurons decreased NREM sleep. Electrophysiological experiments combined with optogenetics showed a direct inhibitory connection between the terminals of RMTg neurons and midbrain dopaminergic neurons. The bidirectional effects of the RMTg on the sleep–wake cycle were mimicked by the modulation of ventral tegmental area (VTA)/substantia nigra compacta (SNc) dopaminergic neuronal activity using a pharmacogenetic approach. Furthermore, during the 2-hour recovery period following 6-hour sleep deprivation, the amount of NREM sleep in both the lesion and control rats was significantly increased compared with baseline levels; however, only the control rats showed a significant increase in SWA compared with baseline levels. Collectively, our findings reveal an essential role of the RMTg in the promotion of NREM sleep and homeostatic regulation.

## Author summary

Sleep–wake behavior is controlled by networks of neurons and neurotransmitters in the brain. There are multiple populations of wake-promoting neurons, but few sleep-promoting neurons have been identified. In this study, we revealed that the rostromedial tegmental nucleus, the GABAergic tail of the ventral tegmental area, regulates non-rapid eye movement sleep. We show that neurons in the rat rostromedial tegmental nucleus, when activated by pharmacogenetics, increase and deepen non-rapid eye movement sleep. Inhibition of these neurons exhibits the opposite effects. Furthermore, rats with lesion in the rostromedial tegmental nucleus have a reduced response of sleep homeostasis following sleep deprivation. We show that stimulation of the terminals of the neurons in the rostromedial tegmental nucleus inhibits dopaminergic neurons in the midbrain. Interestingly, inhibition of these dopaminergic neurons also has sleep-promoting effects. The current results provide a potential target for prolonging non-rapid eye movement sleep, improving sleep quality, and treating sleep disorders in dopamine-implicated mental illness.

## Introduction

Dopamine (DA) produced by neurons in the midbrain plays a key role in processing reward, aversive, and cognitive signals [1]. Abnormal DA is closely associated with neuropsychiatric disorders such as Parkinson disease, schizophrenia, and substance abuse. Severe sleep disturbances have been observed in nearly all of these types of patients [2–5]. Growing evidence suggests that DA-containing neurons are important for arousal maintenance in both humans [6, 7] and animals [8–11]. Moreover, it has been found that activation of ventral tegmental area (VTA) γ-amino-butyric acid (GABA) neurons, which indirectly inhibits the firing of VTA DAergic neurons, is sufficient to elicit a place aversion [12, 13]. This suggests that the ability of midbrain DAergic neurons to regulate sleep–wake behavior and sleep problems in DA-associated mental illnesses may be affected by upstream inhibitory neuronal systems.

The rostromedial tegmental nucleus (RMTg) is a newly identified structure in the brainstem that is rich in μ-opioid receptors. It primarily comprises GABAergic neurons that are distributed dorsolateral to the interpeduncular nucleus (IPN). The RMTg is strikingly innervated by the afferent input from the lateral habenula and additional inputs from the extended amygdala and other closely connected regions, such as the lateral septum and periaqueductal gray matter. The GABAergic axons from the RMTg densely project to midbrain DAergic neurons [14–18]. The RMTg acts as a hub converging and integrating widespread signals toward DAergic systems [19]. Neuroanatomical and electron microscopic studies have found that most RMTg axons form symmetric synapses with tyrosine hydroxylase (TH)-containing dendrites in the substantia nigra compacta (SNc) and VTA [20, 21]. An in vivo electrophysiological study showed that the RMTg exerted greater inhibition of the SNc DAergic neurons than the inhibitory afferents arising from the striatum, globus pallidus, or substantia nigra parsreticulata [20, 22, 23]. Likewise, GABAergic RMTg neurons inhibited the activity of VTA DAergic cells by inhibiting synaptic transmission more effectively than intermediate GABAergic neurons in the VTA [21, 24, 25]. DAergic cells are controlled by excitatory and inhibitory inputs whose balance finely tunes cell activity [26]. The RMTg is now recognized as a GABA brake for midbrain DAergic systems [19].

Aside from the heavy output to the midbrain DAergic neurons, the RMTg sends projections to the dorsal raphe nucleus (DRN), pedunculopontine tegmental and laterodorsal tegmental nuclei (PPT, LDT), and locus coeruleus (LC) and has relatively meager output to the forebrain, including the lateral hypothalamus and lateral preoptic area [14, 15]. Generally, neurons in these cell groups fire most actively during wakefulness [7].

Although the RMTg has been confirmed to inhibit the electrical activity of midbrain DAergic neurons associated with wakefulness activation, whether it is implicated in sleep–wake behavior is unknown. Considering that RMTg neurons are prominently GABAergic and are thus speculated to inhibit rather than facilitate the activity of targeted neurons, we propose that the RMTg is involved in promoting sleep. To test this hypothesis, we employed pharmacogenetics using designer receptors exclusively activated by designer drugs (DREADDs) [27] and pharmacological approaches to manipulate neuronal activity, neurochemistry, electrophysiology, and immunohistochemistry along with optogenetics and transgenic mice to investigate whether the RMTg plays a role in the regulation of sleep and homeostasis. We then explored whether the RMTg nucleus controls sleep through the modulation of DAergic neuron activity.

## Results

### Activation of RMTg neurons by hM3Dq promoted non-rapid eye movement (NREM) sleep in rats

**Fig 1:**
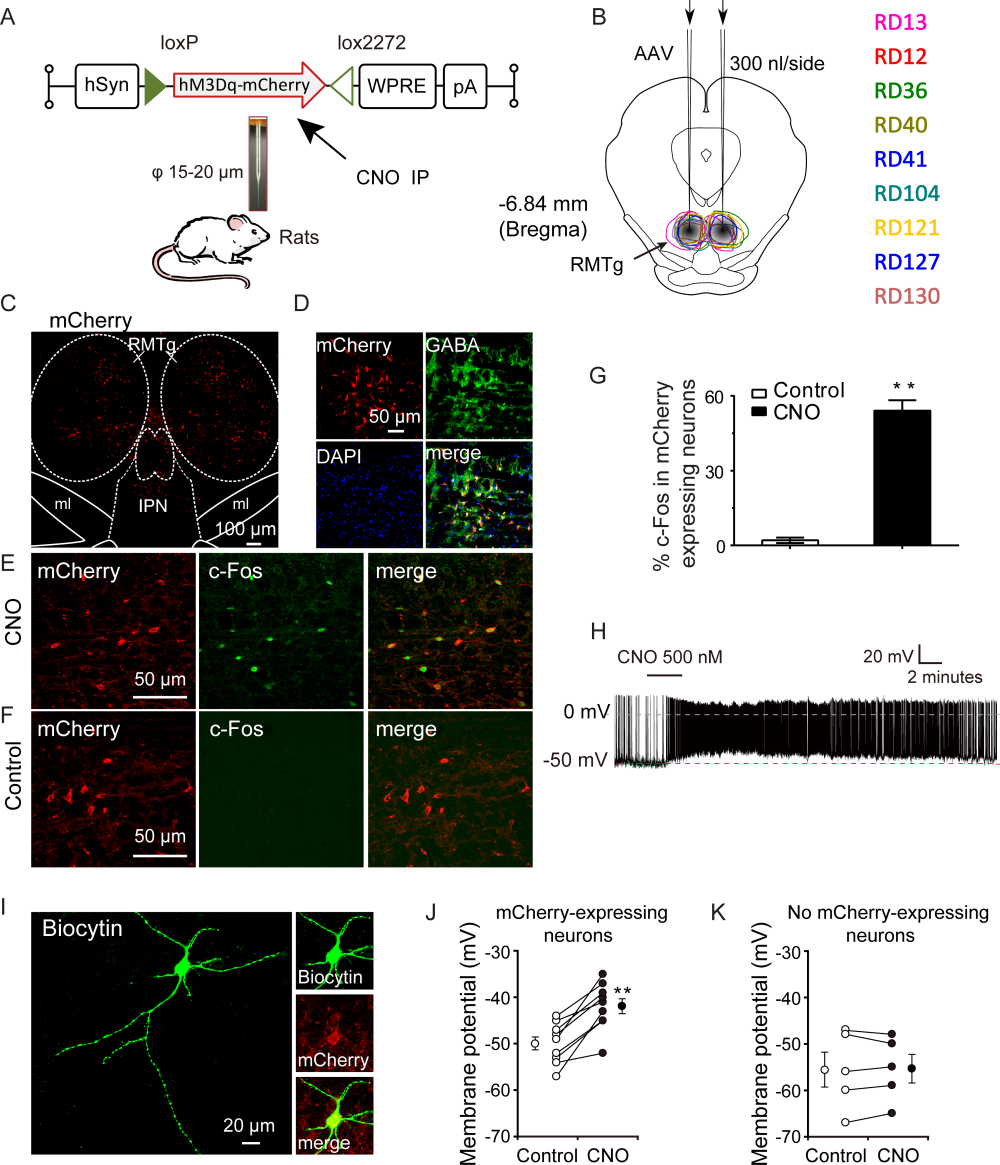
Pharmacogenetic activation of the hM3Dq-expressing rat rostromedial tegmental nucleus (RMTg) neurons. Schematic representation of the Cre-independent adeno-associated virus (AAV) vectors expressing hM3Dq receptors under the control of hSyn promoter. hSyn, human synapsin promoter; WPRE, woodchuck hepatitis virus post-transcriptional regulatory element; CNO, clozapine-N-oxide; IP, intraperitoneal. (B) The coronal section shows the superimposed virus-injected area in 9 rats numbered with the same color characters as the closed curves. The bilateral shaded areas indicate the rat RMTg locations. (C) mCherry immunolabeling (red) indicates hM3Dq-expressing neurons of the RMTg. IPN, interpeduncular nucleus; ml, medial lemniscus. (D) Representative photomicrographs of the RMTg depicting mCherry expression (red), γ-amino butyric acid (GABA) immunoreactivity (green), 4,6-diamidino-2-phenylindole (DAPI, blue), and merge (yellow) images from a rat microinjected with Cre-independent AAV vectors containing hM3Dq. (E, F) CNO (E), not saline (F), drives c-Fos expression in hM3Dq-expressing neurons in the RMTg. (G) Quantification of c-Fos staining in mCherry-expressing RMTg neurons after saline or CNO injection in rats. **p < 0.01 versus saline by unpaired *t* tests (*n* = 3, per group). (H) A typical example of voltage trace recorded from an hM3Dq^+^ rat RMTg neuron during application of CNO in a brain slice. CNO application (horizontal bar) increased the action potential firing. (I) Merged image of mCherry and biocytin showing a fluorescent view of the patched cell. Left panel: biocytin-labeled neuron; right panel: cell with green (biocytin; top), red (hM3Dq-mCherry; middle), and yellow (merged; bottom) fluorescence. (J, K) Average membrane potential of RMTg hM3Dq^+^ neurons (J, *n* = 7 cells), but not hM3Dq^−^ neurons (K, *n* = 5 cells), was significantly increased by CNO application. The results from each cell are shown on the scatterplot. **p < 0.01 versus saline by paired *t* tests. Underlying data can be found in S1 Data.

To test the effects of RMTg neuron activation on sleep and waking regulation, adeno-associated virus (AAV) vectors containing excitatory modified muscarinic G protein-coupled receptors (hM3Dq) (Fig 1A) were bilaterally microinjected into the rat RMTg (Fig 1B). Cell-surface expression of hM3Dq receptors was observed via red fluorescent mCherry protein. Confocal images of double labeling with mCherry and GABA immunofluorescence showed that 87% of mCherry-positive neurons in the RMTg region coexpressed GABA (245 of 280), which indicated GABAergic neurons in the RMTg were targeted. Moreover, we found 56% of GABA-positive neurons colabeled hM3Dq/mCherry (245 of 437) (Fig 1C and 1D), indicating that the virus was efficient for RMTg neurons.

Immunohistochemistry showed that clozapine-N-oxide (CNO, 0.3 mg/kg), a specific hM3Dq agonist (Fig 1E), but not saline (Fig 1F), could drive c-Fos expression in hM3Dq-expressing neurons in the RMTg. Confocal image of double labeling with mCherry and c-Fos immunofluorescence showed that the 56% of mCherry-positive neurons in the RMTg region expressed c-Fos in the CNO group compared to 2% in the saline group (Fig 1G). In addition, bath application of CNO (500 nM) depolarized the RMTg hM3Dq-expressing neurons and significantly increased the firing of action potentials in hM3Dq/mCherry-positive neurons, as indicated by whole-cell current clamp recordings (Fig 1H–1K). Thus, the DREADD system used in this study stimulates the activity of rat RMTg neurons both in vivo and in vitro.

**Fig 2:**
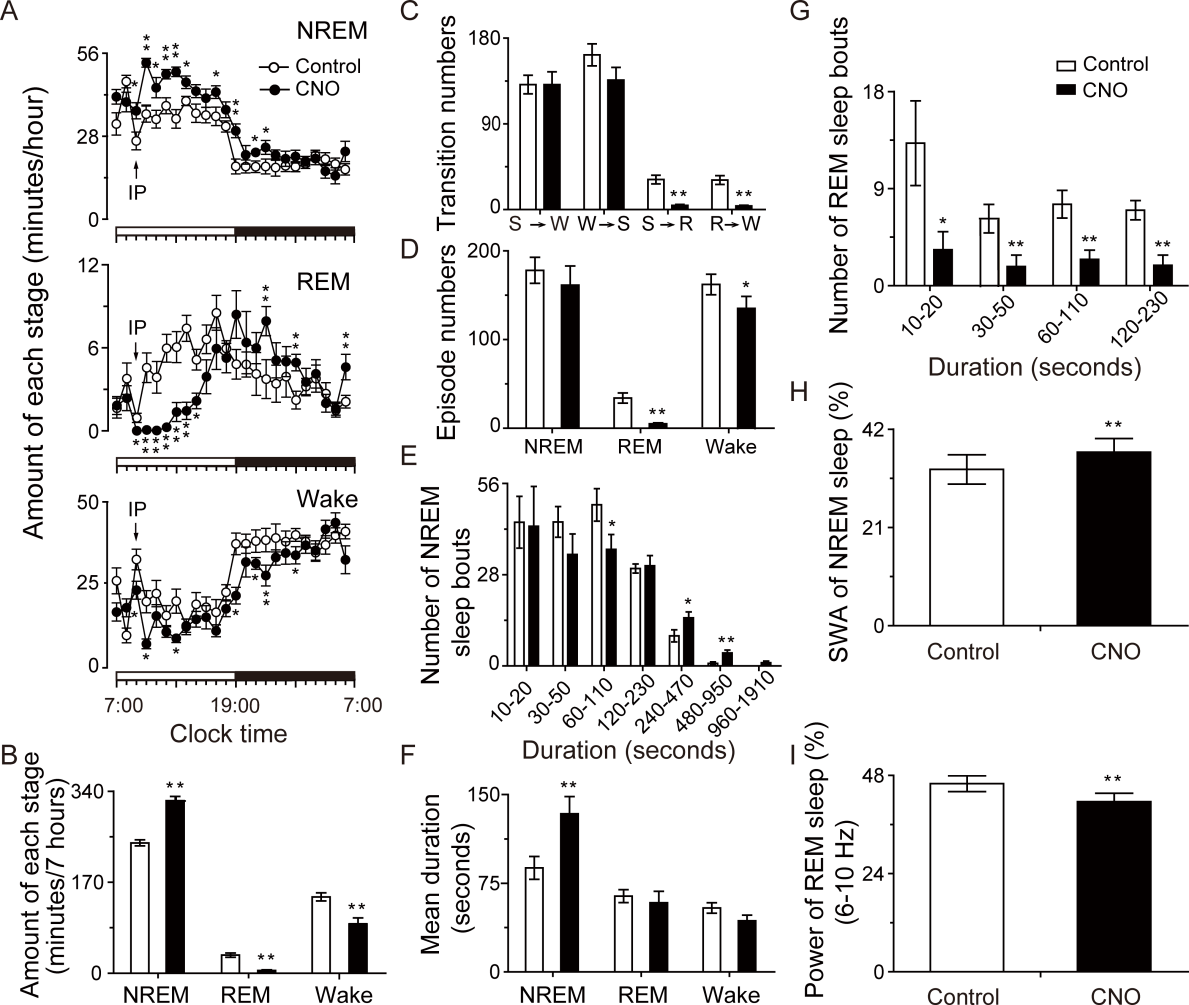
Pharmacogenetic activation of rostromedial tegmental nucleus (RMTg) neurons increased non-rapid eye movement (NREM) sleep in rats. (A, B) Sleep–wake quantities following saline and clozapine-N-oxide (CNO) injection, including average hourly (A) and total sleep–wake amounts (B) during the post-injection period (09:00–16:00 hours). (C–G) Sleep–wake architecture during the post-injection period (09:00–16:00 hours), including conversions between S (NREM sleep), W (wakefulness), and R (REM sleep) stages (C), episode numbers of sleep–wake stages (D), number of NREM and REM sleep bouts with different duration (E, G), and mean duration of sleep–wake stages (F). (H, I) Average slow-wave activity (SWA) of NREM sleep (H) and electroencephalogram (EEG) power of REM sleep in the frequency range of 6–10 Hz (I) during the post-injection period (09:00–16:00 hours) following saline and CNO injection. *p < 0.05, **p < 0.01 versus saline by paired *t* tests. CNO (0.3 mg/kg) was given by intraperitoneal (IP) injection at 09:00 hours (*n* = 9). The horizontal open and filled bars on the *x*-axes indicate the 12-hour light period and the 12-hour dark period, respectively. Underlying data can be found in S1 Data.

On average, the CNO-injected rats showed a 32.2% increase in non-rapid eye movement (NREM) sleep and reductions of 84.6% and 34.8% in rapid eye movement (REM) sleep and wakefulness, respectively, during the 7-hour post-CNO injection period (Fig 2A and 2B). The number of stage conversions from wakefulness to NREM sleep (Fig 2C) and the total NREM sleep episodes (Fig 2D) did not change, even though CNO induced fewer NREM sleep bouts of 1–2 minutes. However, the number of prolonged NREM sleep episodes with durations between 4–16 minutes was significantly increased (Fig 2E), resulting in a longer mean duration of NREM sleep (Fig 2F). CNO administration promoted NREM sleep at the expense of REM sleep, significantly reducing REM sleep bouts of all durations (Fig 2G). Electroencephalogram (EEG) power spectrum analysis revealed that during the 7 hours after CNO injection, the average slow-wave activity (SWA)—a commonly used quantitative measure of sleep intensity, indicated by an EEG power between 0.5 and 4 Hz within NREM sleep [28–30]—in CNO-injected rats increased by 11.0% compared with the saline-injected controls. In contrast, the CNO-injected rats displayed a 9.6% decrease in the power density of REM sleep during the theta band range of 6–10 Hz (Fig 2H and 2I).

The above results indicate that activation of RMTg neurons played an important role in maintaining NREM sleep in rats.

In the saline group, a total of 8 c-Fos^+^ neurons were found in the RMTg of rats infected with viral vectors encoding hM3Dq, indicating that RMTg neurons were not remarkably activated during the spontaneous sleep–wake cycle. When we analyzed distribution of the c-Fos^+^ cells in the CNO group, we found that 78% of them expressed hM3Dq/mCherry (151 of 194) and that the other 22% were mCherry^−^ (43 of 194). These c-Fos^+^/mCherry^−^ neurons may possibly be activated by CNO-induced sleep. Since the regulation of cell activity is very complicated, the increased RMTg c-Fos expression induced by sleep promotion must be a comprehensive result, in which many neural circuits are involved.

### Optogenetic stimulation of RMTg terminals inhibited midbrain DAergic neurons

**Fig 3:**
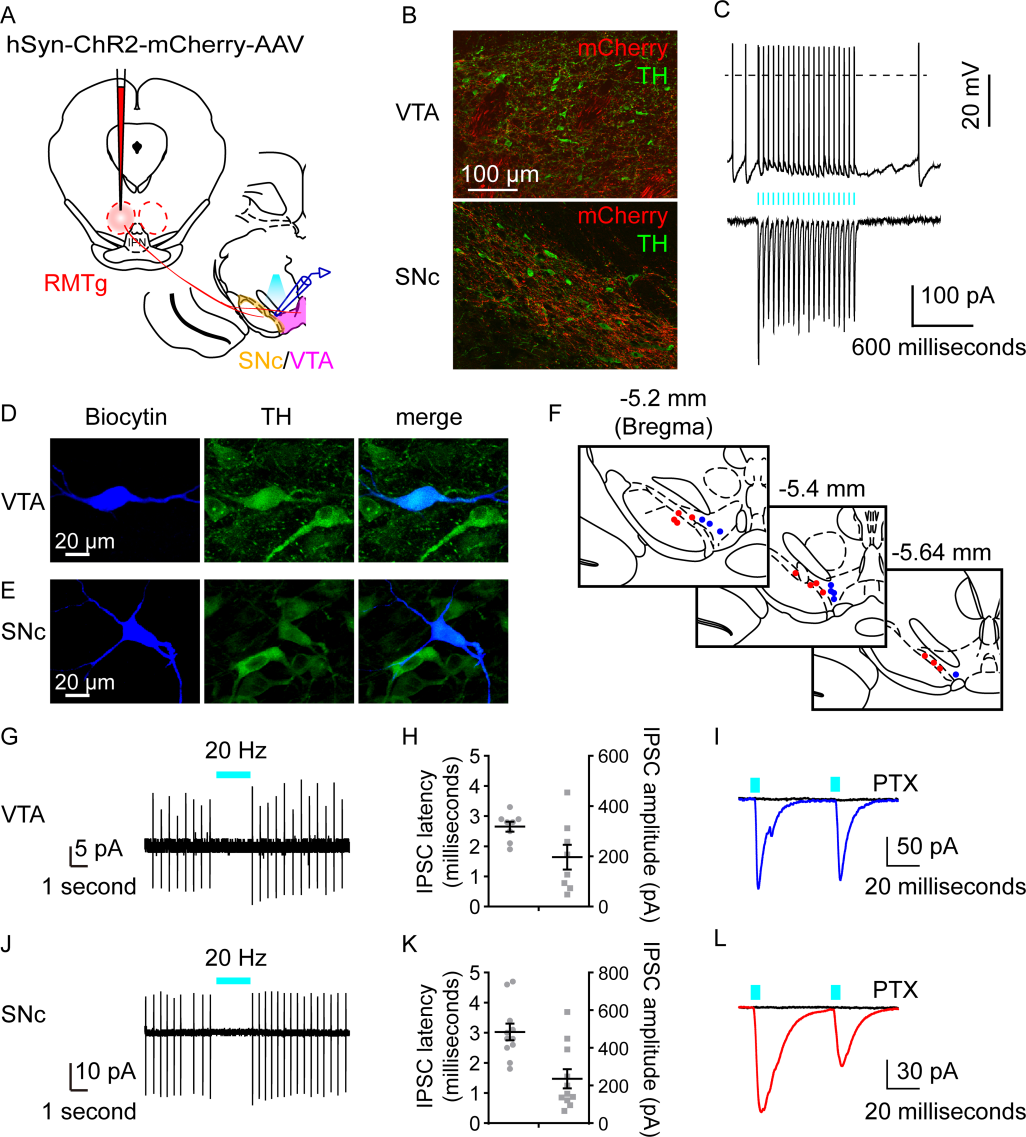
Optogenetic activation of terminals from rostromedial tegmental nucleus (RMTg) neurons inhibited the firing of midbrain dopaminergic neurons. (A) Schematic of the experiment. Adeno-associated virus–channelrhodopsin-2 (AAV-ChR2) was injected into the RMTg of Sprague–Dawley rats, and responses were recorded in the midbrain (ventral tegmental area [VTA]/substantia nigra compacta [SNc]). (B) Representative images of dense mCherry^+^ terminals (red) of RMTg neurons in VTA (top) and SNc (bottom). Tyrosine hydroxylase (TH, green) immunostaining was used to demarcate the VTA and SNc. (C) ChR2-mCherry expressing neurons showed fidelity of spiking (top) following 20 Hz blue light illumination in current-clamp mode. This depolarization coincided with inward current recorded under voltage clamp (bottom). (D, E) Fluorescence micrographs showing a recorded biocytin-filled neuron in the VTA (D) or the SNc (E) that expressed TH. (F) The location of connected biocytin-filled neurons across the rostrocaudal extent of the VTA (blue dots) or the SNc (red dots). (G, J) A typical cell-attached patch recording showed that brief blue light pulses totally suppressed spiking of DAergic neurons in the VTA (G) or SNc (J). (H, K) Latency (left axis) and amplitude (right axis) of light-evoked inhibitory postsynaptic currents (IPSCs) in the VTA (H) or SNc (K) TH-positive neurons (*n* = 8 cells for VTA or 11 cells for SNc from 6 rats). (I, L) Typical traces of postsynaptic currents from a VTA neuron (I) or a SNc neuron (L) evoked by blue light. The evoked currents were completely abolished by application of picrotoxin (PTX) (100 μM; black lines). Underlying data can be found in S1 Data.

To explore the functional nature of the RMTg-to-midbrain DAergic connections, we employed an optogenetic-assisted circuit mapping approach. Channelrhodopsin-2 (ChR2), a blue light-gated cation channel, was expressed in RMTg neurons by injecting AAV-ChR2-mCherry into the RMTg of Sprague–Dawley rats. After 3 weeks, acute coronal brain slices containing the RMTg or VTA/SNc were prepared for in vitro patch-clamp recording (Fig 3A). The expression of ChR2 allowed the activation of cell bodies within the RMTg and the selective stimulation of terminals from the RMTg that projected to the VTA and SNc.

First, we found that there were dense mCherry^+^ terminals of RMTg neurons in VTA and SNc, which showed anatomical connections between RMTg neurons and midbrain DAergic neurons (Fig 3B). We then tested the responses of ChR2-expressing neurons within the RMTg to optogenetic stimulation. Blue light pulses at 20 Hz evoked action potentials with high fidelity and elicited robust photocurrents under voltage mode (Fig 3C). Next, cells in the VTA and the SNc were patch clamped while blue light flashes were used to stimulate the axon terminals of RMTg neurons. To identify the cell types of recorded midbrain neurons, we added biocytin to the pipette solution and performed immunostaining using TH as a marker for DAergic neurons after recording. We found that light-evoked inhibition could be recorded in TH-positive neurons within the VTA and the SNc (Fig 3D and 3E), and the connected neurons were distributed throughout the rostrocaudal extent of the VTA/SNc (Fig 3F). In the cell-attached patch mode, photostimulation (5-millisecond pulses, 20 Hz) of RMTg terminals in the VTA or the SNc was sufficient to decrease the firing rate; in some cases, light application totally inhibited the spikes of midbrain neurons, and the firing rate recovered immediately upon the termination of photostimulation (Fig 3G and 3J). In the whole-cell voltage-clamp mode, light evoked fast inhibitory postsynaptic currents (IPSCs) in VTA and SNc TH-positive neurons with latencies less than 5 milliseconds in both cases (Fig 3H and 3K), indicating a direct inhibitory connection between the terminals of RMTg neurons and midbrain TH-positive neurons. Moreover, the light-evoked IPSCs were completely abolished by 100 μM picrotoxin (PTX, a GABA_A_R antagonist; Fig 3I and 3L), indicating that these responses were mediated by GABA released from axon terminals of RMTg neurons.

### Sleep promotion induced by RMTg activation was mimicked by inhibition of VTA/SNc DAergic neurons in TH-Cre mice

The present study confirmed that midbrain DAergic neurons receive direct inhibitory innervation from RMTg neurons, which is now known to be the predominant GABAergic control for midbrain DAergic neuron activity. Furthermore, functional studies have revealed that the roles of the RMTg are mediated by the modulation of DAergic neurons. Thus, we wondered whether sleep control by the RMTg also occurs through the modulation of DAergic neuron activity.

**Fig 4:**
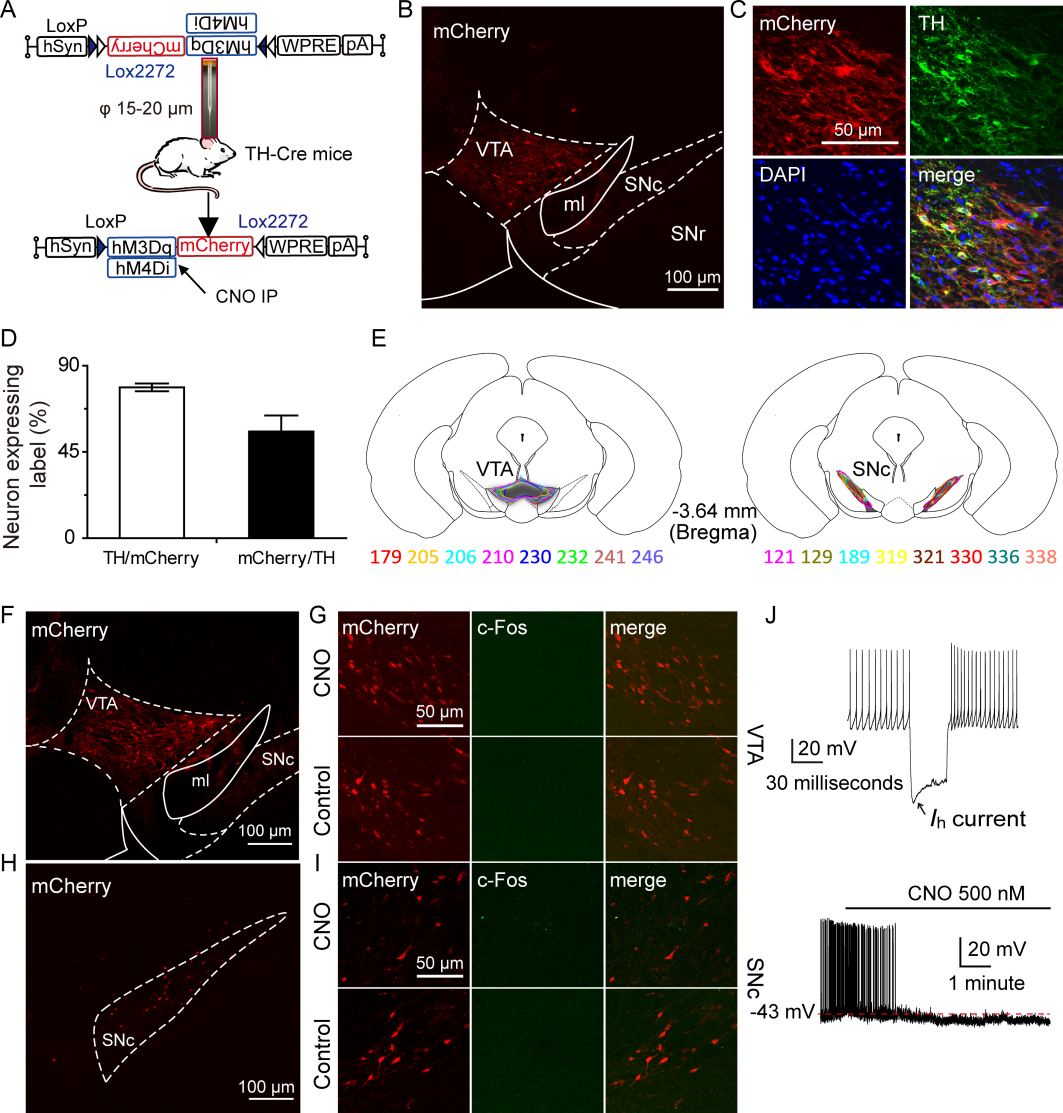
Pharmacogenetic inhibition of ventral tegmental area (VTA)/substantia nigra compacta (SNc) dopaminergic (DAergic) neurons in tyrosine hydroxylase (TH)-Cre mice. Schematic representation of Cre-dependent adeno-associated virus (AAV) vectors expressing hM3Dq/hM4Di-mCherry under the control of human synapsin (hSyn) promoter. WPRE, woodchuck hepatitis virus post-transcriptional regulatory element; CNO, clozapine-N-oxide; IP, intraperitoneal. (B) Microinjection of AAV-DIO-hM4Di-mCherry into the VTA induced hM4Di expression (red). ml, medial lemniscus; SNc, substantia nigra, compacta; SNr, substantia nigra, reticular part. (C) Representative photomicrographs of the VTA depicting mCherry expression (red), TH (green), and 4,6-diamidino-2-phenylindole (DAPI, blue) immunoreactivity and merge images (yellow) from a TH-Cre mouse microinjected with Cre-dependent AAV vectors containing hM4Di. (D) Statistics of the coexpression of TH and mCherry immunofluorescence of VTA neurons (*n* = 3, per group). (E) Drawings of superimposed AAV-injected area in the VTA (left, *n* = 8) and SNc (right, *n* = 8) of TH-Cre mice with different colors. (F, H) Representative photomicrographs of mCherry expression after microinjection of AAVs containing hM4Di into the VTA (F) and SNc (H). (G, I) CNO (G, I; top) and saline (G, I; bottom) did not induce c-Fos expression in hM4Di-expressing DAergic neurons in VTA (G) or SNc (I). (J) In TH-Cre mice, the firing property of a recorded VTA hM4Di/mCherry-positive DAergic neuron with hyperpolarization-activated cation current (*I*_h_) (top) and CNO perfusion inhibited its firing rate (bottom). Underlying data can be found in S1 Data.

To specifically manipulate DAergic neuron activity, Cre-dependent AAVs (Fig 4A) were microinjected into the VTA or SNc areas (Fig 4E) of TH-Cre mice to express the sequence of hM3Dq or hM4Di, which was activated by CNO treatment; as a result, the DAergic neurons were reversibly excited or inhibited, respectively. To detect whether AAVs could be expressed specifically in DAergic neurons rather than in other kinds of neurons, hM4Di-expressing AAVs combined with red fluorescent protein mCherry were microinjected into the VTA of TH-Cre mice. Immunofluorescent staining of colocalization (yellow) of mCherry (red), DAPI (blue), and TH (green) showed that 56% of TH-expressing neurons in the VTA coexpressed hM4Di/mCherry (170 of 302) and that 79% of hM4Di/mCherry-positive neurons were colabeled with TH (170 of 214) (Fig 4B–4D), which validated the efficiency and specificity of this targeting strategy for midbrain DAergic neurons.

To test the brain states in which the DAergic neurons were inhibited, we microinjected Cre-inducible AAVs expressing hM4Di fused with red fluorescent protein into the target of TH-Cre mice. The microinjection sites and AAV-hM4Di-infected areas were confirmed by mCherry expression in the VTA (Fig 4F) and SNc (Fig 4H). Intraperitoneal injection of both CNO and saline did not induce c-Fos expression in hM4Di-expressing mice (Fig 4G and 4I). Next, we performed in vitro electrophysiological experiments to confirm the inhibitory effects of CNO on hM4Di-expressing neuron activity. The recorded mCherry-expressing neuron displayed firing properties with hyperpolarization-activated cation current (*I*_h_) (Fig 4J, top) that were similar to the properties previously reported for DAergic neurons [31]. Whole-cell current clamp recordings demonstrated that bath application of CNO (500 nM) inhibited the firing rate of a VTA hM4Di-expressing DAergic neuron (Fig 4J, bottom). These results indicated that after AAV-hM4Di microinjection, DAergic neurons were inhibited by CNO in vivo and in vitro.

**Fig 5:**
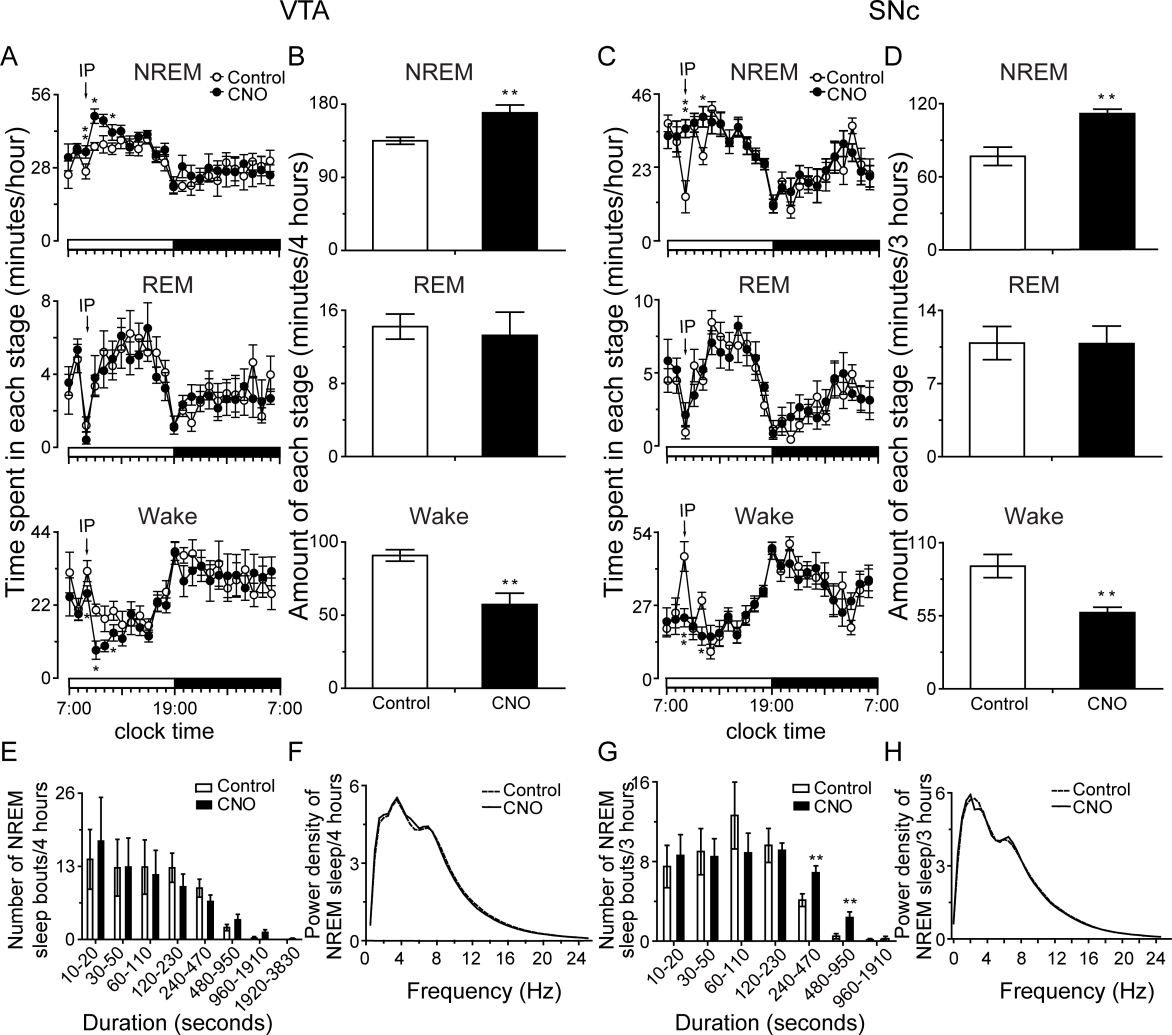
Pharmacogenetic inhibition of ventral tegmental area (VTA)/substantia nigra compacta (SNc) dopaminergic (DAergic) neurons promoted non-rapid eye movement (NREM) sleep in tyrosine hydroxylase (TH)-Cre mice. (A, C) Time course changes of NREM and REM sleep and wakefulness after administration of saline or clozapine-N-oxide (CNO) in hM4Di-expressing TH-Cre mice in the VTA (A, *n* = 8) and SNc (C, *n* = 8) DAergic neurons. (B, D) Total sleep–wake amounts during 09:00–13:00 hours (B, VTA) or 09:00–12:00 hours (D, SNc) after administration of saline or CNO. (E–H) Number of NREM sleep bouts with different duration (E, G) and average power density of NREM sleep (F, H) in 4 hours (E, F, VTA) or 3 hours (G, H, SNc) after saline or CNO injection in TH-Cre mice. *p < 0.05, **p < 0.01 versus saline by paired *t* tests. CNO (1.0 mg/kg) was given by intraperitoneal (IP) injection at 09:00 hours. The horizontal open and filled bars on the *x*-axes indicate the 12-hour light period and the 12-hour dark period, respectively. Underlying data can be found in S1 Data.

When CNO was administered at 09:00 hours, the TH-Cre mice microinjected with AAV-hM4Di into the VTA or SNc showed an increase in NREM sleep, a decrease in wakefulness, and no change in REM sleep. Compared with the saline control, when VTA DAergic neurons were inhibited, total NREM sleep at 4 hours following CNO treatment was increased by 25.6%, while wakefulness decreased by 36.9% (Fig 5A and 5B). Similarly, the inhibition of SNc DAergic neurons by CNO administration induced a 3-hour increase of 45.5% in NREM sleep, with a 37.8% decrease in wakefulness (Fig 5C and 5D). The number of prolonged NREM sleep episodes with durations between 8 and 64 minutes showed a tendency of increase, which may have caused the increase of NREM sleep when VTA DAergic neurons were inhibited using the pharmacogenetic approach (Fig 5E). Similarly, inhibition of SNc DAergic neurons produced an increase in the number of NREM sleep episodes lasting between 4 and 16 minutes (Fig 5G). Although the amount of NREM sleep induced by pharmacogenetic activation of the RMTg was mimicked by inhibiting midbrain DAergic neurons, the enhanced quality of NREM sleep with higher SWA levels induced by activation of the RMTg was not demonstrated (Fig 5F and 5H).

### Sleep–wake effects of RMTg lesions in rats

**Fig 6:**
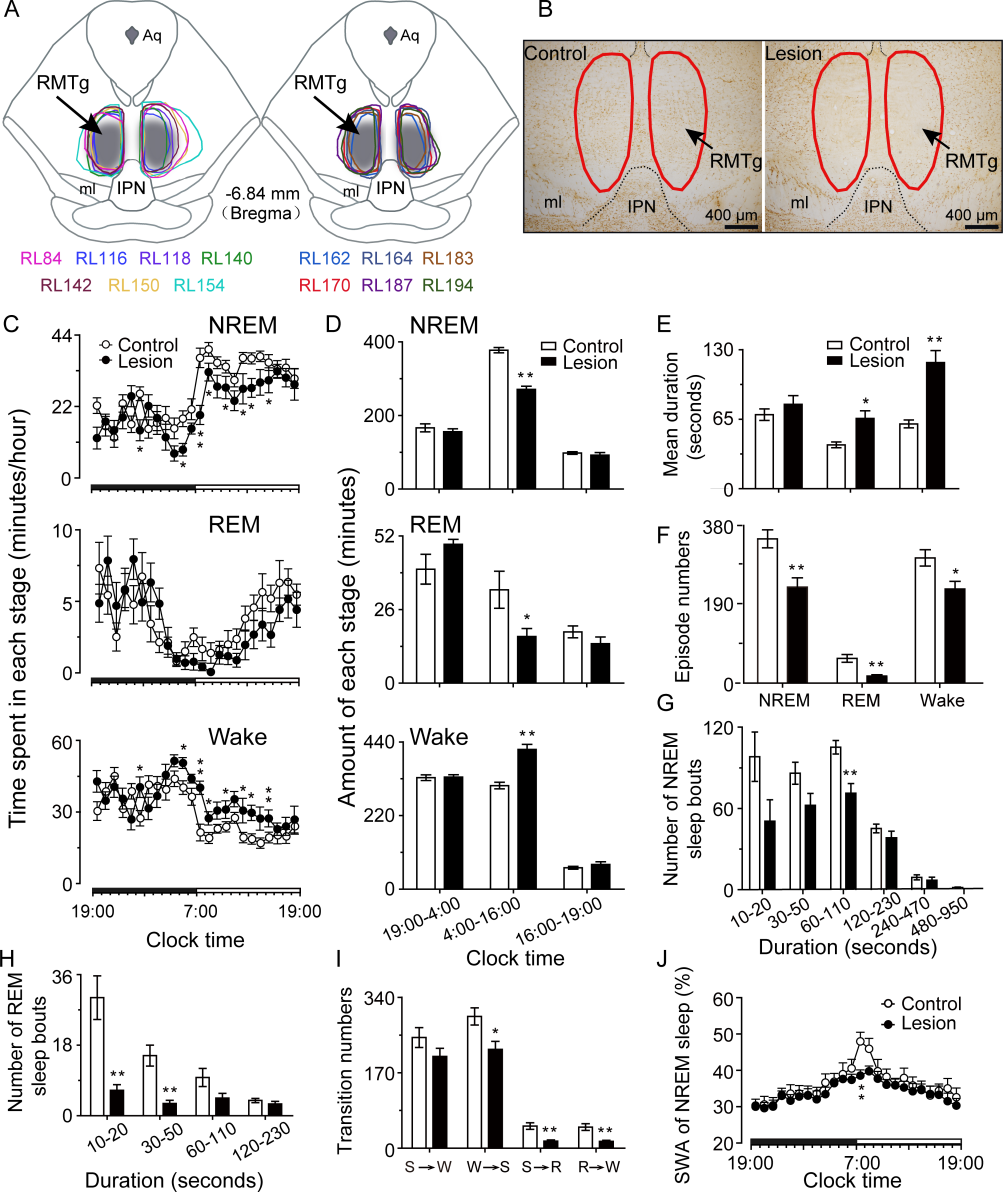
Sleep and wake profiles on day 8 after inducing rostromedial tegmental nucleus (RMTg) lesions with ibotenic acid. (A) Diagrammatic drawing of lesion extent with ibotenic acid in the total 13 lesioned animals whose electroencephalograms (EEGs) were successfully collected after 8 days (RL84, RL116, RL140, RL142, RL162, RL164, RL183, and RL170, *n* = 8), 16 days (RL140, RL142, RL164, RL170, RL150, RL154, RL187, and RL194, *n* = 8), 25 days (RL116, RL164, RL183, RL187, RL194, and RL118, *n* = 6), and 8 days following 6-hour sleep deprivation (RL162, RL164, RL183, and RL170, *n* = 4) of lesions. The lesioned rats were numbered with the same color characters as the closed curves. The bilateral shaded areas indicate the RMTg area. (B) Photographs of representative coronal sections from a control (left) and a lesioned (right) rat by NeuN staining. The parts with red circles show the location of the RMTg. IPN, interpeduncular nucleus; ml, medial lemniscus. (C) Hourly amount of non-rapid eye movement (NREM) and rapid eye movement (REM) sleep and wakefulness. (D) Total sleep–wake amount during the 9-hour dark period (19:00–04:00 hours), the subsequent 12-hour period (04:00–16:00 hours) and the 3-hour light period (16:00–19:00 h). (E–I) Sleep–wake architecture during 04:00 to 16:00 hours. Mean duration (E) and total episode numbers (F) of NREM and REM sleep and wakefulness; episode spectrum of NREM (G) and REM (H) sleep; transitions between S (NREM sleep), W (wakefulness), and R (REM sleep) stages (I). (J) Hourly slow-wave activity (SWA) (expressed as percentage of delta power [0.5–4 Hz] in NREM sleep per hour) of NREM sleep. *p < 0.05, **p < 0.01 versus controls. Control (*n* = 9); lesion (*n* = 8). The horizontal filled and open bars on the *x*-axes indicate the 12-hour dark period and the 12-hour light period, respectively. Underlying data can be found in S1 Data.

In order to investigate whether the RMTg plays a role in physiological sleep promotion, bilateral lesions were formed in the RMTg neurons by microinjection of ibotenic acid, a cell-specific neurochemical toxin, using a glass microelectrode technique. After a recovery period, continuous EEG was performed for 48 hours. We compared lesioned and saline-injected control animals for sleep–wake parameters, including time spent in each sleep–wake state, bout numbers, average bout durations, and SWA. On completion of EEG recordings, the lesion position and extent was immunohistologically confirmed by neuron-specific nuclear-binding protein (NeuN) staining. Compared with the intact neurons in the control rats, the animals with ibotenic acid-induced lesions showed extensive cell loss within the RMTg. The lesion extent of each rat was outlined by drawing the boundary of the NeuN-positive neuron population when the staining images had been scaled by the same proportion as the referenced atlas in the same canvas (Fig 6A and 6B).

At 8 days after lesions, rats showed a 25.2% decrease in NREM sleep and a 47.8% decrease in REM sleep along with a 35.9% increase in wakefulness during the 12-hour period from 04:00 to 16:00 hours (Fig 6C and 6D). Analysis of sleep architecture showed that the average duration of NREM sleep was not shortened, and the REM sleep duration was prolonged in the lesioned rats from 04:00 to 16:00 hours. However, the total number of episodes of NREM and REM sleep was markedly decreased in the lesioned rats (Fig 6E and 6F). The rats with RMTg lesions tended to have fewer NREM sleep bouts in the range of 10–50 seconds than controls, although the difference was not statistically significant, and had significantly fewer NREM sleep bouts between 1–2 minutes and REM sleep bouts between 10–50 seconds than controls (Fig 6G and 6H). The lesioned rats also had fewer conversions from wakefulness to NREM sleep, NREM sleep to REM sleep, and REM sleep to wakefulness (Fig 6I). Therefore, the decreased NREM and REM sleep amount was mainly the result of a reduction in the number of short fragments of NREM sleep (<2 minutes) and REM sleep (<1 minute) in the lesioned rats. Across the 24-hour period, SWA was at the highest level—48.0% ± 2.5% and 38.6% ± 1.6% in control and lesioned animals, respectively—during 07:00–08:00, immediately after light onset. The highest level of SWA in the NREM sleep was significantly lower in the lesioned rats than in the control rats (*p* < 0.01). Then, SWA gradually decreased until immediately before and after lights off, when it was at the lowest level (Fig 6J).

Similarly, at 16 days after RMTg lesions, the amount of sleep and wakefulness and sleep–wake architecture differed significantly between the lesion and control groups. NREM sleep decreased by 9.4%, and REM sleep decreased by 14.3%, with corresponding increases in wakefulness by 10.7% was observed in the lesioned rats in comparison with the controls over a 24-hour period (see S1A and S1B Fig). The lesioned rats had fewer episodes of NREM and REM sleep and fewer conversions from wakefulness to NREM sleep, NREM sleep to REM sleep, and REM sleep to wakefulness than the control rats (see S1C–S1E Fig). SWA in NREM sleep during the hour immediately after lights turned on was also significantly lower in the lesioned rats (42.1% ± 2.3%) than in the control rats (50.6% ± 2.9%) (see S1F Fig).

The loss of NREM and REM sleep in the RMTg lesion rats was not observed at 25 days after neuron damage (see S2A and S2B Fig). There were no differences in episode bouts, durations, or conversions among NREM and REM sleep and wakefulness between the lesioned and control rats (see S2C–S2E Fig). However, at 25 days after RMTg lesions, the rats still had lower SWA levels in NREM sleep, similar to those at 8 and 16 days. SWA in NREM sleep during the first hour of the light phase (34.9% ± 6.4%) was continually and markedly lower than that of the control rats (48.0% ± 2.0%) (see S2F Fig).

The results suggest that the RMTg is involved in the initiation of NREM sleep, which is compensated by some brain structures at 25 days after damage to RMTg neurons. Furthermore, the RMTg maintains NREM sleep depth, and this effect lasts longer than that on sleep initiation.

### Effects of RMTg lesions on homeostatic regulation of sleep

**Fig 7:**
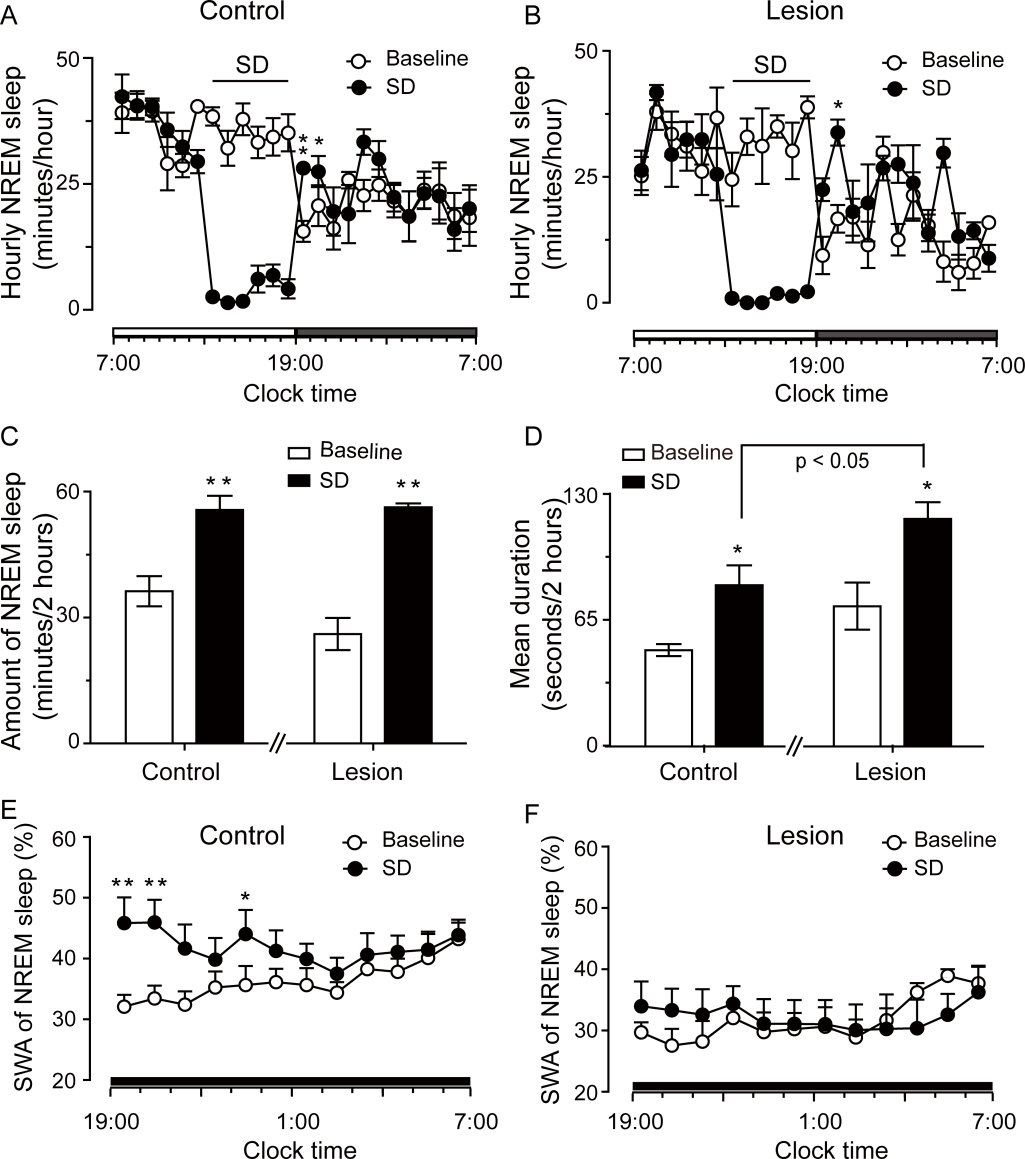
Rats with RMTg lesions by ibotenic acid for 8 days showed a deficit in rebound of non-rapid eye movement (NREM) sleep quality in response to 6-hour sleep deprivation (SD). (A, B) Time course of NREM sleep in control (A) and RMTg lesioned rats (B). Sleep was deprived from 13:00 to 19:00 hours. (C, D) Amount of NREM sleep (C) and mean duration of NREM sleep (D) during 19:00–21:00 hours following 6-hour SD. (E, F) Time course of slow-wave activity (SWA) (expressed as percentage of delta power [0.5–4 Hz] in NREM sleep per hour) from 19:00 to 07:00 hours following 6-hour SD in control (E) and RMTg lesioned rats (F). *p < 0.05, **p < 0.01 versus corresponding baseline using 2-way ANOVA followed by paired *t* tests. Control (*n* = 6); lesion (*n* = 4). The horizontal open and filled bars on the *x*-axes indicate the 12-hour light and the 12-hour dark period, respectively. Underlying data can be found in S1 Data.

Sleep homeostasis is primarily studied through sleep deprivation (SD) experiments [32]. Thus, we performed 6-hour SD from 13:00 to 19:00 hours in control and lesioned rats and compared their rebound sleep to determine the role of the RMTg in homeostatic regulation of sleep. The control rats and rats with 8 days of RMTg lesions exhibited similar responses in their NREM sleep during the subsequent recovery period following SD (Fig 7A and 7B). We calculated the total time spent in NREM sleep for 2 hours after SD because the increase in NREM sleep was maintained for 2 hours during the recovery period after 6-hour SD. From 19:00 to 21:00 hours, there was no difference between the control and lesioned rats in the baseline level of NREM sleep, which increased by 57.6% and 128.2%, respectively, after 6-hour SD (Fig 7C). In both groups, the increase in NREM sleep was mainly due to prolonged bout duration (Fig 7D).

To compare the rebound of SWA within NREM sleep after 6-hour SD in the control and lesioned groups, the SWA data were analyzed using 2-way ANOVA followed by paired *t* tests. The analysis revealed a significant increase in SWA in the control rats during the first 7 hours (from 19:00 to 02:00 hours) following 6-hour SD (*p* = 0.041) compared with the baseline level. In contrast, 6-hour SD induced no increase in SWA in the lesioned rats even during the first 2 hours (from 19:00 to 21:00 hours) immediately after SD (*p* = 0.394) compared with the baseline level. There was no change from baseline in the mean SWA in control (33.1% ± 2.2%) or lesioned (27.5% ± 2.7%) rats during the first 2-hour interval from 19:00 to 21:00 hours. After 6-hour SD, the average SWA increased by 38.2% ± 4.3% and 21.5% ± 4.2% from the baseline levels to 46.1% ± 4.0% and 33.5% ± 3.7% in the control and lesioned rats, respectively (Fig 7E and 7F). The magnitude of the increase in SWA after 6-hour SD was significantly lower in lesioned rats than in control rats (*p* < 0.05, unpaired *t* test).

The above results showed that in the recovery period following 6-hour SD in RMTg lesioned rats, the amount of NREM sleep rebounded normally, but the rebound of NREM sleep depth was impaired. Therefore, the RMTg plays an important role in the homeostatic regulation of NREM sleep.

### Effects of local inhibition of RMTg neurons on sleep–wake behavior in rats

**Fig 8:**
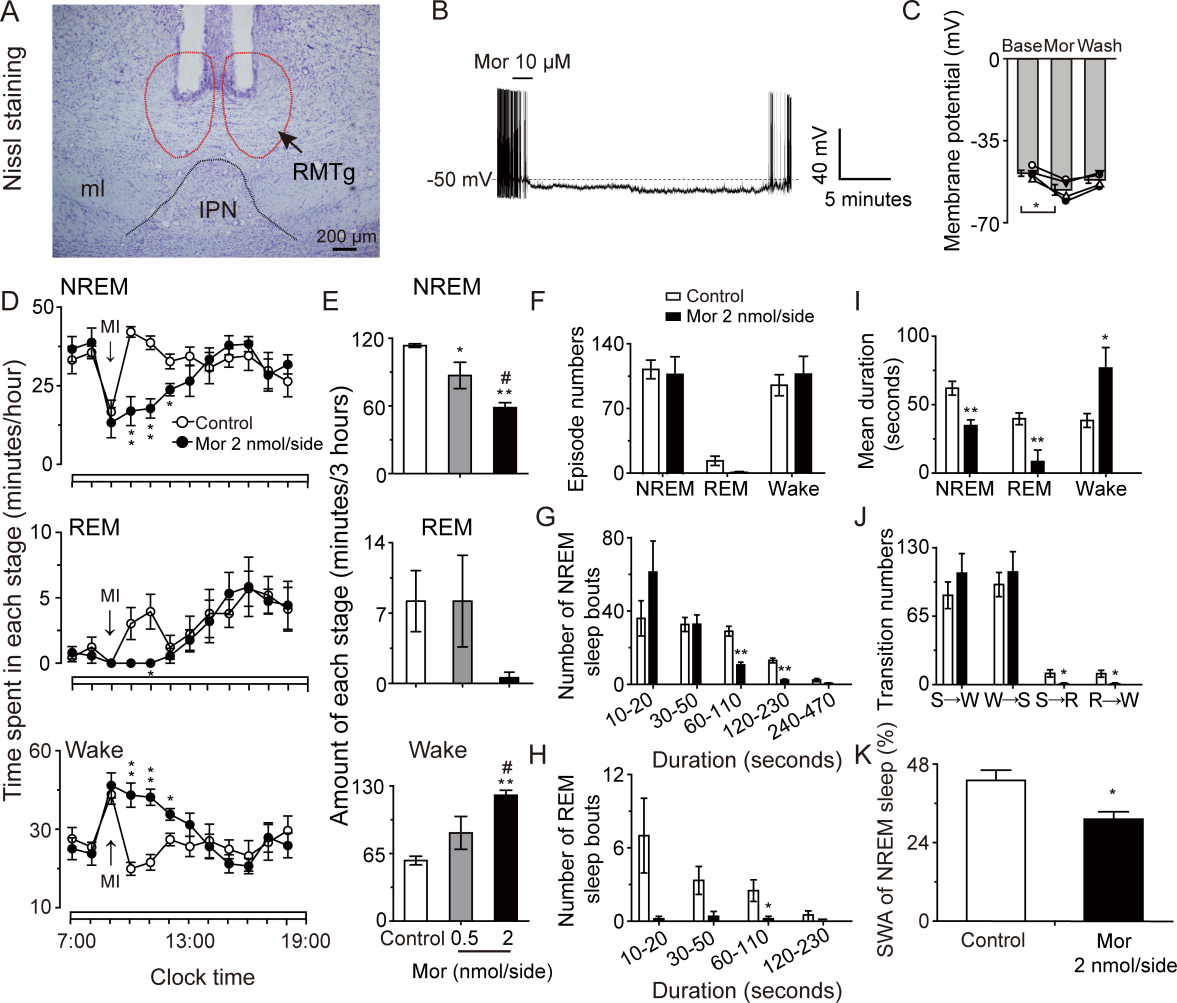
Microinjection of morphine in rat rostromedial tegmental nucleus (RMTg) decreased non-rapid eye movement (NREM) sleep in a dose-dependent manner. (A) Nissl staining showed the implantation sites of the guide cannulas. (B, C) Morphine inhibited RMTg neurons in vitro. A typical trace showed that morphine induced hyperpolarization and inhibited spontaneous firing of a neuron in the RMTg (B) and changes of membrane potentials of RMTg neurons under whole-cell current-clamp with application of morphine. *n* = 4 cells, *p < 0.05 versus baseline using 1-way ANOVA followed by least significant difference (LSD) (C). (D) Time course of NREM and REM sleep and wakefulness during the light period (07:00–19:00 hours) in rats treated with artificial cerebrospinal fluid (ACSF) or morphine at 09:00 hour. (E) Sleep–wake quantities following ACSF or morphine microinjection in rats during post-injection period (10:00–13:00 hours). *p < 0.05, **p < 0.01 versus ACSF and #*p* < 0.05 versus 0.5 nmol/side morphine using 1-way ANOVA followed by LSD. (F–K) During the post-injection period (10:00–13:00 hours) after ACSF or morphine microinjection in rats, episode numbers of each stage (F); number of NREM (G) and REM (H) sleep bouts with different duration; mean duration of each stage (I); stage transitions between S (NREM sleep), W (wakefulness), and R (REM sleep) stages (J); and slow-wave activity (SWA) of NREM sleep (K). *p < 0.05, **p < 0.01 versus ACSF using unpaired *t* tests. MI, microinjection; Mor, morphine. ACSF (*n* = 6); morphine 0.5 nmol/side (*n* = 5); morphine 2 nmol/side (*n* = 5). The horizontal open bars on the *x*-axes indicate the 12-hour light period. Underlying data can be found in S1 Data.

The RMTg is rich in μ receptors, and in vivo electrophysiological experiments have found that morphine, a μ-receptor agonist, inactivates RMTg neurons [25, 33]. Therefore, to rapidly and reversibly inhibit RMTg neurons, we bilaterally microinjected morphine through guide cannulas. After EEG recording, Nissl staining was performed to confirm the microinjection site. Only samples in which the needle tips were located in the RMTg were included in data analysis (Fig 8A). Under current-clamp conditions, morphine perfusion at 10 μmol/L (μM) was found to decrease the firing rate and induce significant hyperpolarization of RMTg neurons from −49.3 ± 1.3 to −56.3 ± 2.2 mV (*p* < 0.05). In some cases, morphine completely inhibited the firing of RMTg neurons within 1–2 minutes (Fig 8B and 8C). These results were consistent with previous reports that opioids could inactivate RMTg neurons [33].

During the 3-hour post-microinjection of morphine at 2 nmol/side, the rats showed an immediate 48.5% decrease in NREM sleep and a 93.1% decrease in REM sleep, with an increase in wakefulness by 107.6% compared with the artificial cerebrospinal fluid (ACSF) controls (Fig 8D and 8E). EEG architecture analysis showed that during the 3 hours after microinjection of morphine at 2 nmol/side, although the episode numbers of NREM and REM sleep did not change (Fig 8F), the longer fragments of NREM sleep with durations between 1 and 4 minutes and longer fragments of REM sleep between 1 and 2 minutes were significantly decreased (Fig 8G and 8H). As a result, the mean duration of NREM and REM sleep was shortened compared with ACSF microinjection controls (Fig 8I). In addition, the morphine-induced inhibition of RMTg neurons did not affect the number of stage shifts between wakefulness and NREM sleep (Fig 8J). EEG power spectrum analysis revealed that the power density of NREM sleep was significantly decreased in the rats microinjected in the RMTg with 2 nmol/side morphine compared with the ACSF controls over the frequency range of delta activity from 0.5 to 4 Hz during the 3 hours from 10:00 to 13:00 hours following microinjection (*p* < 0.05) (Fig 8K). The above results suggest that the RMTg plays roles in the maintenance of the duration and quality of NREM sleep.

Similar results were observed after pharmacogenetic inhibition of cells in the RMTg with Gi-coupled DREADDs (see S3 Fig). When RMTg neurons were inactivated (see S4A and S4B Fig), the rats showed a decrease in time spent in NREM sleep and a corresponding increase in time awake, but no decrease in REM sleep (see S4C Fig). The decreased NREM sleep resulted mainly from the reduction of NREM bout occurrence, particularly the number of shorter episodes with durations between 10–110 seconds (see S4D and S4E Fig) and fewer conversions from wakefulness to NREM sleep (see S4F Fig) following CNO injections. These results suggest that the RMTg plays a role in the initiation of NREM sleep. However, the changes in the mean duration and SWA level of NREM sleep did not differ significantly between saline-treated rats and CNO-treated rats (see S4G and S4H Fig). The decrease in NREM sleep without any change in SWA induced by pharmacogenetic inhibition of the RMTg using hM4Di was mimicked by selective pharmacogenetic activation of VTA/SNc DAergic neurons using hM3Dq in TH-Cre mice (see S5 Fig).

## Discussion

Using pharmacogenetic activation and neurotoxic lesions, as well as microinfusion and pharmacogenetic inactivation approaches, the present study found that RMTg neurons were essential for physiological NREM sleep. After RMTg neurons were damaged, the rats had reduced NREM sleep, which was mainly due to the decreased number of short-duration bouts and fewer transitions from wakefulness to NREM sleep. The results suggest that the RMTg is involved in the initiation of NREM sleep. Moreover, the RMTg mediates the maintenance of NREM sleep quality. SWA in the NREM sleep of the control and lesioned rats was prominent at lights on, when sleep pressure is highest, and gradually decreased as sleep progressed, which was in line with previous reports [30, 32]. However, the highest level of SWA in the RMTg-lesioned rats was significantly and continuously weaker than that in the control animals. NREM sleep quality was impaired by RMTg lesions, and the impairment lasted longer than the reduction in the amount of NREM sleep, which returned to baseline at 25 days after lesions. Therefore, the RMTg is essential to maintaining SWA at the peak level in NREM sleep; in contrast, its role in maintaining NREM sleep quantity can be fulfilled by other brain regions.

Although lesion formation is a widely used approach in neuroscience research, it has some limitations, such as causing irreversible damage to neurons and fibers of passage, which is not a natural physiological situation, and as a result, it is not possible to regulate neuronal activity reversibly [34]. Moreover, functional recovery may be seen, and the effects of lesions are confounded by secondary compensatory changes in response to the damage [35]. Complementary to the research on permanent neurotoxic lesions, temporary inactivation studies will help to clarify the engagement of the RMTg in the physiological regulation of sleep. We observed that the inactivation of RMTg neurons by both intra-RMTg injections of morphine and the Gi-coupled DREADD approach markedly reduced NREM sleep duration, which confirms the necessity of RMTg for the control of NREM sleep. The present study also shows that the normal neuronal activities of RMTg are necessary for the maintenance of NREM sleep, as demonstrated by the reduction in the numbers of longer bouts of NREM sleep with morphine-RMTg injection, as well as for the initiation of NREM sleep, as shown by the decreased numbers of short-duration bouts of NREM sleep and fewer transitions from wakefulness to NREM sleep with Gi-coupled DREADDs and neurotoxic lesions. The role of the RMTg in the promotion of NREM sleep is suggested again by the pronounced increase in NREM sleep duration with higher SWA levels after pharmacogenetic activation of RMTg neurons.

Most likely, the different effects of the RMTg on sleep architecture and sleep depth arise from differences in pharmacological and pharmacogenetic effects. The lesion method is an excellent approach, but it does not exclude a neuromodulatory role. Another conventional pharmacological intervention of microinfusion as well as the pharmacogenetic method with Gi-coupled AAV vectors that has developed rapidly over the past decade can be used to inactivate neurons. Intra-RMTg injection of morphine inhibits neurons by stimulating μ-opioid receptors and is likely to inhibit many more cells within the nucleus and diffuse to adjacent nontargeted areas, whereas the DREADD method is mainly used in the local nucleus to inhibit modified muscarinic receptors (M-Rs), especially with lower doses of AAVs, and generally produces mild effects. Moreover, the downstream signaling pathways of morphine and CNO (ligand for M-Rs) are also different.

It is worth noting that the vast majority of mCherry-positive cells in our study were confined to the RMTg (see S6 Fig). However, some labeled cells were found ventrally in the IPN. It has been reported that lesions of the bilateral fasciculus retroflexus, a major input to the IPN, result in reduced REM sleep [36]. Another study found that the Gscl (a homeobox transcription factor, Goosecoid-like) knockout mice exhibited a decrease in REM sleep and an increase in NREM sleep [37]. These findings indicate that the normal function of IPN is required for wakefulness and REM sleep. The neighboring dorsal and median raphe also had a small number of infected neurons. The main neurons in dorsal and median raphe are serotonergic neurons, which are sufficient for inducing wakefulness. Drugs that increase serotonin tone, such as selective serotonin reuptake inhibitors, generally increase wakefulness in both humans and rodents [38]. In all, the effects of the expression of hM3Dq/hM4Di spread outward somewhat are not linked to the NREM sleep promotion of RMTg neurons.

Sleep is an inactive state, characterized by the cessation of locomotion, reduced responsiveness, and easy reversibility [28]. From this point of view, the present results were in agreement with Lavezzi et al., who found that the activation of RMTg neurons through bilateral infusions of the GABA_A_ receptor antagonist bicuculline suppressed locomotor activity in rats [39].

REM sleep was also inhibited during CNO-induced NREM sleep in rats with DREADD activation of RMTg neurons. Why REM sleep was so strongly inhibited is unclear, but one explanation may be that the effect of NREM sleep-promotion is too pronounced to be completely compensated by wakefulness alone. In a previous study, similar REM sleep reduction was observed when activation of the parafacial zone markedly induced NREM sleep [40]. It is also possible that RMTg GABAergic neurons project to and inhibit REM sleep-promoting PPT and LDT neurons [38]. However, considering that lesions or inactivation of the rat RMTg did not markedly affect REM sleep quantity, we feel that the RMTg does not play an important role in the physiological control of REM sleep.

Homeostatic control of sleep, 1 of 2 sleep regulation processes, is the increased propensity for sleep during prolonged wakefulness [41]. Animals may compensate for sleep loss by an increased amount of NREM sleep and SWA in NREM sleep [28]. Our study showed that 6-hour SD induced a 2-hour increase in NREM sleep, which was similar to previous studies, which found that NREM sleep was significantly above the corresponding baseline in the first 2-hour interval after 6-hour SD [42, 43]. However, in the present study, NREM sleep intensity significantly increased in control rats, whereas the RMTg-lesioned rats showed a reduced response of sleep homeostasis, as indicated by the lack of enhanced delta EEG power in NREM sleep after 6-hour SD. Several previous studies showed the same results. Rats with nucleus accumbens core lesions showed reductions in SWA rebound, but not in sleep duration response, after 6-hour SD [43], and the A_1_-R (a kind of adenosine receptor) knockout mice responded similarly following 3-hour SD [44].

SWA in NREM sleep is considered to be functionally involved in sleep–wake homeostasis. However, the neuronal mechanisms and neurochemical factors that drive these homeostatic responses are largely unknown [7, 32]. Studies have suggested that SWA is generated in the cerebral cortex and thalamus [38], and the basal forebrain is another important structure for sleep homeostasis [45]. The DRN and PPT nucleus are important target nuclei of RMTg [15]. Inhibition of DRN neurons by inhibition of calmodulin-dependent kinase II or damage with 3,4-methylenedioxymethamphetamine was found to significantly enhance NREM sleep and delta power density during NREM sleep [46, 47]. In addition, activation of cholinergic LDT neurons suppressed slow EEG activity during NREM sleep [48]. Moreover, the VTA and SNc, which are major strong outputs of RMTg, have efferent and afferent connections with the DRN, LDT, locus coeruleus, lateral and posterior hypothalamus, basal forebrain, and thalamus [49]. Collectively, the downstream nuclei may be involved in the circuit through which the RMTg regulates sleep homeostasis. Further studies are needed to clarify the inconsistency of RMTg’s effects on the 2 aspects of homeostasis regulation.

RMTg is widely recognized as a brake for the DAergic system. Early electrophysiological findings, which showed that VTA and SNc DAergic neurons do not change their firing rates across the sleep–wake cycle [50, 51] and that there is no change in the time spent in electrocortical waking in animals with electrolytic lesions of the catecholamine-containing neurons of the VTA and SNc [52], suggest that DAergic neurons are not involved in the regulation of sleep–wake states [7]. Meanwhile, some evidence suggests that DA signals are associated with sleep promotion [49, 53]. Since DAergic neurons are mainly distributed in the VTA and SNc, we directly manipulated the activity of midbrain DAergic neurons using pharmacogenetic methods to observe their effects on sleep–wake behavior. We found that selective inhibition of VTA/SNc DAergic neurons promoted NREM sleep, which was also observed with activation of RMTg neurons. In contrast, selective activation of VTA/SNc DAergic neurons produced an increase in wakefulness. The current results are in agreement with a recent finding that optogenetic stimulation or inhibition of VTA DAergic neurons initiates and maintains wakefulness or increases NREM sleep, respectively [10].

### Conclusion

We find that the RMTg is essential for NREM sleep and homeostatic regulation. Since the RMTg exerts major inhibitory control over the midbrain DAergic system, we hypothesize that the RMTg regulates sleep–wake behavior through the modulation of DAergic neuron activity. However, we cannot exclude the possibility that other RMTg GABAergic projected targets also contribute to the roles of the RMTg in sleep–wake behavior.

For further study, we will use transgenic VGAT (vesicular GABA transporter)-Cre mice to specifically target the GABAergic neurons in order to expand on our findings in rats that RMTg neurons are necessary for sleep and use pharmacogenetic and optogenetic manipulations together with polysomnographic recordings to elucidate the role of the downstream elements of GABAergic RMTg neurons in the control of sleep.

There is evidence that sleep, especially NREM sleep with high levels of EEG delta power, is important not only for neurobehavioral functions such as memory consolidation [54] but also for peripheral physiological functions such as the maintenance of normal glucose homeostasis [55, 56]. Thus, the current results suggest that the RMTg may be considered as a potential intervention for prolonging NREM sleep duration and improving sleep quality to maintain human health. Moreover, the data obtained in the present study enrich our understanding of how the brain regulates sleep–wake behavior and provide a potential target for understanding the roles of DA in the physiological regulation of sleep–wake states as well as in the pathologic process of sleep disturbances. Our results will inform the development of potential therapeutic targets against sleep disorders in DA-implicated mental illness.

## Methods

### Ethics statement

All experimental procedures involving animals were approved by the Committee on the Ethics of Animal Experiments of School of Basic Medical Sciences, Fudan University, with license identification number 20150119–067. The animals were anesthetized with an IP injection of chloral hydrate before surgery or killing. The animals were put on the heating pad until they woke up from anesthesia after surgery. During the postoperative recovery period, the animals were observed every day, and the sawdust was kept clean. Every effort was made to minimize animal suffering or discomfort and to reduce the number of animals used.

### Animals

Male Sprague–Dawley rats (280–370 g) were obtained from the Laboratory Animal Center, Chinese Academy of Sciences (Shanghai, China). Adult male TH-Cre mice and non-Cre-expressing littermate mice (8–16 weeks old, 25–30 g) were also used. The animals were housed in individual cages at an ambient temperature (22 ± 0.5°C) with relative humidity of 60% ± 2% in an automatically controlled 12:12-hour light/dark cycle (lights on at 07:00 hours, illumination intensity approximately 100 lux), with free access to food and water.

### AAV generation

The AAVs of serotype rh10 for AAV-hSyn-DIO-hM3Dq-mCherry, AAV-hSyn-DIO-hM4Di-mCherry, and AAV-hSyn-DIO-ChR2-mCherry were generated by tripartite transfection (AAV-rep2/caprh10 expression plasmid, adenovirus helper plasmid, and pAAV plasmid) into 293A cells. After 3 days, the 293A cells were resuspended in ACSF, freeze-thawed 4 times, and treated with benzonase nuclease (Millipore) to degrade all forms of DNA and RNA. Subsequently, the cell debris was removed by centrifugation, and the virus titer in the supernatant was determined using an AAVpro Titration Kit for Real Time PCR (Takara). The final viral concentrations of the transgenes were 1 × 10^12^–2 × 10^12^ genome copies/mL. Aliquots of viral vectors were stored at −80°C before stereotaxic injection.

### Surgery

To induce lesions in the RMTg, rats were anesthetized with chloral hydrate (10% in saline, 360 mg/kg) and immobilized in a Stereotaxic Alignment System (RWD Life Science, Shenzhen, China). Ibotenic acid (1% in saline, 200 nL/side; Sigma, St. Louis, Missouri, United States) was bilaterally injected through a glass pipette (glass stock: 1 mm in diameter; tip: 10–20 μm) with nitrogen gas pulses of 20–40 psi using an air compression system [57] into the RMTg according to the atlas [58] (coordinate relative to bregma: AP −6.8 mm; ML ± 0.3 mm; DV −8.4 mm) for 5–10 minutes. After leaving the pipette in the brain for an additional 5 minutes, the pipette was slowly retracted. Control rats received 200 nL/side saline.

For microinjection of morphine in the RMTg, rats were implanted with 2 guide cannula (30 gauge). The cannulas were inserted at stereotaxic coordinates based on the rat brain atlas [58] (coordinate relative to bregma: AP −6.8 mm; ML ± 0.3 mm; DV −7.4 mm). The 2 cannulas were fixed to the skull with dental cement and 3 stainless steel screws for anchorage [59].

In order to manipulate neuronal activity, we used hM3Dq or hM4Di that was selectively activated or inhibited by the pharmacologically inert agent CNO [60]. Under anesthesia with chloral hydrate (10% in saline, 360 mg/kg), a burr hole was made, and a fine glass pipette containing AAVs carrying Cre-independent hM3Dq, hM4Di, or ChR2 was lowered bilaterally into the rat RMTg (coordinate relative to bregma: AP −6.8 mm; ML ± 0.3 mm; DV −8.4 mm). The AAV vectors were delivered with 300 nL/side.

Mice were anesthetized with chloral hydrate (5% in saline, 720 mg/kg) and then placed in a stereotaxic frame so that the head was fixed. After opening a burr hole, a fine glass pipette containing AAV carrying Cre-dependent hM3Dq/hM4Di (Taiting, Shanghai, China) was bilaterally lowered into the VTA (coordinate relative to bregma: AP −3.4 mm; ML ± 0.3 mm; DV −4.0 mm) or SNc (coordinate relative to bregma: AP −3.4 mm; ML ± 1.2 mm; DV −4.0 mm) according to the atlas[5]. The AAV vectors (50 nL/side) were delivered over a 5-minute period per hemisphere. After an additional 10 minutes, the pipette was slowly withdrawn.

Following ibotenic acid or saline injection (or guide cannula implantation) or after 2 weeks of recovery from virus injection, the rats were implanted with EEG and electromyography (EMG) electrodes for polysomnographic recordings. The implant consisted of 2 stainless steel screws (1-mm diameter) inserted through the frontal (AP +2 mm; ML +3 mm) and parietal (AP −4 mm; ML +3 mm) bones, and a stainless steel screw (1.5-mm diameter) inserted in the left frontal bone (AP +3 mm; ML −3 mm) as a reference electrode. All of the above positions were coordinately relative to the bregma [58]. The mice were recovered for 2 weeks after virus injection before electrodes were implanted. Two stainless steel screws (1-mm diameter) were inserted through the skull into the cortex (AP +1 mm to the bregma; AP +1 mm to the lambda; ML +1.5 mm to the midline) [61] and served as EEG electrodes.

Two Teflon-coated, stainless steel wires were bilaterally placed into both trapezius muscles for EMG recordings in rats or mice. All electrodes for the rats or mice were attached to a connector and fixed to the skull with dental cement. The animals were then allowed to recover on a heating pad until awakening from anesthesia [62].

### Sleep recording and vigilance state analysis

After a 1-week recovery period from EEG electrode implantation, the animals were transferred to the recording room and habituated to the recording cables and conditions for 2–3 days. Following this habituation period, 48 hours of EEG/EMG recordings were performed on all the animals. The data collected during the first 24 hours served as baseline, and the second 24 hours served as experimental data.

Morphine hydrochloride, a nonselective μ-opioid receptor agonist, diluted in ACSF, was injected at 09:00 hours through a syringe connected with a lengthened flexible pipe under red illumination; thus, the rats could freely move or have rest without irritant body touch, disturbing noises, and natural light. A syringe that was designed with an oblique tip for easy aligning was gently and smoothly inserted into the hollow and straight guide cannula (O.D. = 0.6 mm). A volume of 0.5 μL morphine was injected in each side by microinjector at a slow and constant speed for 0.5 minutes. Injection was made unilaterally in sequence. After the end of each injection, the syringe was left for an additional 3 minutes for complete local absorption. The control group was injected with ACSF.

In the DREADD experiment, saline was administered IP at 09:00 hours on day 1 of the EEG recording. On the next day, CNO (LKT Laboratories, Minneapolis, Minnesota, US) was dissolved in saline before use and injected at 09:00 hours at 1 mg/kg for mice (0.1 mL/10 g) or 0.3 mg/kg for rats (1 mL/100 g).

Cortical EEG and EMG signals were amplified, filtered (EEG, 0.5–30 Hz; EMG, 20–200 Hz), digitized at a sampling rate of 128 Hz, and recorded using VitalRecorder (Kissei Comtec, Nagano, Japan). When complete, polygraphic recordings were automatically scored offline by 10-second epochs as waking, NREM sleep, and REM sleep using SleepSign according to standard criteria. Defined sleep–wake stages were examined visually and corrected if necessary [57].

### SD

SD was achieved by gentle handling that included tapping the cage, introducing novel objects into the cage, or removing the rat from the cage when behavioral signs of sleep were observed. Rats were deprived of sleep during the light phase for 6 hours from 13:00 to 19:00 hours. Undisturbed rats that served as a control group were never disturbed when they were spontaneously awake, feeding, or drinking in the same time period as the corresponding 6-hour SD group [30, 42].

### Immunohistochemistry

On completion of EEG recordings of the rats with lesions or morphine microinjection, the rats were anesthetized with chloral hydrate (10% in saline, 360 mg/kg), with 150–200 mL saline followed by 500 mL 4% paraformaldehyde (PFA) in PBS through the heart. The brains were removed, post-fixed for 4–5 hours at 4°C in 4% PFA, and then equilibrated in phosphate buffer containing 10%, 20%, and 30% sucrose solution at 4°C. The brain sections were serially cut in the coronal plane at 30 μm on a freezing microtome (CM1950, Leica, Wetzlar, Germany), protected in cryoprotectant solution, and stored at −20°C until further processing for immunostaining.

For verification of ibotenic-acid-induced brain lesions, one series of tissues was processed for NeuN staining. Brain sections were incubated in primary antibody in PBS containing Tween-20 (PBST) (mouse anti-NeuN, 1:50,000; Millipore, Bedford, Maryland, US) overnight, and then staining was revealed using the avidin–biotin complex method (ABC kit SC-2017; Santa Cruz Biotechnology, Santa Cruz, California, US). The sections were incubated for 2 hours in biotinylated secondary antibody in PBST (1:500), followed by incubation with avidin–biotin–horseradish peroxidase (HRP) conjugate and staining with 3,3-diaminobenzidine tetrahydrochloride (DAB). Sections were then mounted, dried, dehydrated, and cover slipped. Only samples in which the lesion sites were confined to the RMTg were included in data analysis.

For confirmation of the microinjection site of morphine, one series of sections was subjected to Nissl staining. Brain sections were mounted on adhesive slides, dried naturally for 2 consecutive days, and stained by cresyl violet method. Sections were washed in water and PBS successively, incubated in 0.1% cresyl violet for 15 minutes, differentiated in graded ethanol, and cleared in xylene before being cover slipped. All sections containing a cannula-insertion site were compared with a rat brain atlas to confirm the 3-dimensional coordinates of the site relative to bregma. Only experiments in which the tip of the microinjection cannula was located above the RMTg were included in data analysis [59].

The AAV vectors were linked with a red fluorescent protein mCherry; therefore, the viral injection site was determined by mCherry expression. Induction of c-Fos, the protein of an immediate early gene, is supposed to be an indicator of neuronal activity [63]. Thus, whether the virus-infected neurons were activated or inhibited could be determined through c-Fos expression. After EEG/EMG recording, the animals were injected with saline or CNO (0.3 mg/kg for rats; 1 mg/kg for mice). Ninety minutes later, the animals were anesthetized and perfused, and the brain coronal sections were prepared. The brain tissue sections were rinsed in 0.1 M PBS (3 times, 5 minutes/each wash) and then incubated in rabbit anti-c-Fos primary antibody (1:5,000; Millipore) diluted in PBST for 48 hours. The tissues were washed 3 times in PBS and incubated with Alexa Fluor 488-conjugated donkey anti-rabbit secondary antibody in PBST (1:1,000; Invitrogen, Carlsbad, California, US) for 2 hours in the dark. After being washed in PBS, the sections were mounted on glass slides, cover slipped using FluoroGuard Mounting Medium, and kept at 4°C before imaging [40]. Colocalization of mCherry and c-Fos expression was observed by confocal microscopy. Localization of the rat RMTg and mouse VTA and SNc were confirmed by staining and reference to the brain atlas.

For double immunofluorescence staining of TH/mCherry or GABA/mCherry, brain sections were incubated with a rabbit antibody against TH (1:3,000; Millipore) or GABA (1:1,000; Invitrogen) in PBST over night at 4°C. The sections were then rinsed and incubated in a donkey anti-rabbit Alexa Fluor 488-conjugated secondary antibody (1:1,000; Invitrogen) at room temperature for 2 hours in the dark. After 3 washes in PBS, sections were incubated in 4,6-diamidino-2-phenylindole (DAPI; 1:3,000; Invitrogen) for 10 min at room temperature. Finally, sections were washed in PBS and mounted on glass slides using FluoroGuard Mounting Medium.

### Cell counting

For quantification of the colocalization of mCherry-expressing RMTg neurons in rats and VTA/SNc DAergic neurons in mice microinjected with hM3Dq/hM4Di-containing vectors and other histological markers (GABA, c-Fos, TH), the area used for counting was demarcated by cells that were labeled by mCherry. All cell counting was conducted blindly on 3 × 2 tiled confocal images of the target area. For each rat or mouse, brain sections were analyzed bilaterally [64].

### Electrophysiological experiments

To investigate whether CNO manipulates the activity of AAV-infected cells or RMTg has an inhibitory projection to midbrain DAergic neurons, male Sprague–Dawley rats, 20–30 days old weighing 50–60 g, were microinjected with AAV vectors carrying Cre-independent hsyn–hM3Dq (hM4Di, ChR2)-mCherry under anesthesia with chloral hydrate into the RMTg, and TH-Cre mice were microinjected with AAV vectors carrying Cre-dependent hsyn–hM3Dq/ hM4Di–mCherry into the VTA or SNc. Slices containing the RMTg of the rats or VTA/SNc of TH-Cre mice were prepared from the animals 3 weeks after AAV microinjection or from male Sprague–Dawley rats, 20–30 days old weighing 50–60 g, without AAV injection to investigate whether morphine inhibited RMTg neurons in vitro. The RMTg or VTA/SNc was identified according to stereotaxic coordinates [58, 61].

Coronal slices of the rats (280-μm thick) were cut using a vibratome (VT-1200S; Leica) in ice-cold sucrose-based ACSF, bubbled with 95% O_2_ and 5% CO_2_, containing 230 mM sucrose, 2.5 mM KCl, 3 mM MgSO_4_, 1.25 mM NaH_2_PO_4_, 26 mM NaHCO_3_, 0.5 mM CaCl_2_, and 10 mM d-glucose. The slices were allowed to recover for at least 1 hour in a holding chamber with ACSF without sucrose in a water bath (32°C) before recording. For preparation of the mouse brain slices, the coronal slices were cut at 300-μm thickness in ice-cold glycerol-based ACSF, which was different from the rats, containing 260 mM glycerol, 5 mM KCl, 1.25 mM KH_2_PO_4_, 1.3 mM MgSO_4_, 0.5 mM CaCl_2_, 20 mM NaHCO_3_, and 10 mM glucose.

Coronal slices were transferred to the recording chamber, where they were held down with a platinum ring. Carbonated ACSF with 95% O_2_ and 5% CO_2_ flowed through the bath (2 mL/minute). Patch pipettes were pulled from thick-walled borosilicate glass capillaries (1.5-mm outer diameter, 0.84-mm internal diameter, Sutter Instruments, San Rafael, California, US) using a 2-step vertical puller (PC-10; Narishige, Japan). Pipette resistance was typically 4–7 MΩ when filled with internal solution containing 120 mM potassium gluconate, 20 mM KCl, 1 mM MgCl_2_, 0.16 mM CaCl_2_, 10 mM HEPES, 0.5 mM EGTA, 2 mM Mg-ATP, and 0.5 mM NaGTP. RMTg or VTA/SNc neurons were identified under visual guidance using a fixed-stage upright microscope (BX-51; Olympus, Tokyo, Japan) fitted with a 40 × water immersion objective lens. The image was detected with an infrared sensitive charge coupled device camera (U-TV1X-2; Olympus) and displayed on a screen in real time. The output signals were amplified (Molecular Devices, Eugene, Oregon, US), filtered at 5 kHz, and digitized at 20 kHz using a National Instruments digitization board (NI-DAQmx, PCI-6052E; National Instruments, Austin, Texas, US). Neurons were current clamped to record spontaneous action potentials and/or membrane potentials. The series resistance and input resistance were monitored throughout the cell recording, and data were discarded when either of the 2 resistances changed by >20% [57, 65]. To visualize the recorded cells in the RMTg, biocytin (0.2%) was included in the pipette solution to confirm the position of patched cells. Slices were fixed immediately after recording in 4% formaldehyde for 2 hours and then immersed in 0.3% PBST. Slices were incubated in Fluor-488-conjugated streptavidin (Invitrogen, 1:2,000, 12 hours at 4°C). Sections were mounted on slides using FluoroGuard Antifade Reagent (Bio-Rad, Hercules, California, US) and visualized under an Olympus microscope.

For the optogenetic experiment, whole-cell and cell-attached recordings were made from RMTg or VTA/SNc DAergic neurons. ChR2 was stimulated by 473-nm light delivered via an optical fiber coupled to a laser source (Guang Teng, Shanghai, China). For recording light evoked inhibitory synaptic currents, the internal solution contained 105 mM potassium gluconate, 30 mM KCl, 4 mM ATP-Mg, 10 mM phosphocreatine, 0.3 mM EGTA, 0.3 mM GTP-Na, and 10 mM HEPES. In the voltage-clamp mode, cells were held at −70 mV. When needed, 25 μM d-(-)-2-amino-5-phosphonopentanoic acid (d-APV), 5 μM NBQX, and 100 μM PTX were added to block NMDA, AMPA, and GABA_A_ receptors, respectively. The internal solution also contained 0.2% biocytin. To test for expression of TH, brain slices were incubated in rabbit anti-TH antibody (1:2,000, Millipore) containing 3% normal donkey serum (v/v), 0.5% Triton X-100 (v/v) for 24 hours at 4°C. This was followed by incubation with Alexa Fluor 488-conjugated donkey anti-rabbit (1:800; Invitrogen) and Alexa Fluor 405 streptavidin (1:1,000, Invitrogen) for 12 hours at RT.

### Statistical analysis

The sum of sleep and wakefulness and other sleep architecture parameters in the RMTg-lesioned and control rats were compared using unpaired *t* tests. The sleep–wake profiles among groups with different doses of morphine microinjection and vehicles were assessed using 1-way ANOVA followed by least significant difference tests. The hourly durations of each stage and SWA analyses were compared using 2-way ANOVA (repeated measures) followed by unpaired *t* tests. Two-way ANOVA (repeated measures) followed by paired *t* tests were conducted to analyze the sleep rebound induced by 6-hour SD in rats.

For viral microinjection, the sum of sleep and wakefulness and other sleep architecture parameters after CNO or saline injection as well as membrane potentials of rat RMTg neurons before and after bath application of CNO were compared using paired *t* tests. The hourly duration of each stage of sleep and wake profiles was compared using 2-way ANOVA (repeated measures) followed by paired *t* tests.

For GABA, c-Fos, or TH immunohistochemistry analysis, each group consisted of data obtained from 3 rats or TH-Cre mice; as a result, a total of 3 bilateral sections containing target area were analyzed for each animal. The quantification results were compared using unpaired *t* tests between CNO and saline control groups.

All results were expressed as the mean ± SEM. We analyzed the data using Prism 5.0 (GraphPad software, San Diego, California, US). In all cases, *p* < 0.05 was taken as the level of significance.

## Supporting information

S1 Data The excel spreadsheet (in separate sheets) contains the underlying numerical data used to generate all graphs and histograms in the manuscript. (XLSX)

S1 Fig Sleep and wake profiles on day 16 after rostromedial tegmental nucleus (RMTg) lesions with ibotenic acid. (A) Hourly amount of NREM and REM sleep and wakefulness. (B) Total sleep–wake amount during the 12-hour dark (19:00–7:00 hours) and the 12-hour light (07:00–19:00 hours) and the 24-hour period. (C–E) Sleep–wake architecture over 24 hours. Mean duration (C) and episode numbers (D) of NREM and REM sleep and wakefulness; transitions between S (NREM sleep), W (wakefulness), and R (REM sleep) stages (E). (F) Hourly slow-wave activity (SWA) of NREM sleep. *p < 0.05, **p < 0.01 versus control. Control (*n* = 10); lesion (*n* = 8). The horizontal filled and open bars on the x-axes indicate the 12-hour dark period and the 12-hour light period, respectively.
(TIF)

S2 Fig Sleep and wake profiles on day 25 after rostromedial tegmental nucleus (RMTg) lesions with ibotenic acid. (A) Hourly amount of NREM and REM sleep and wakefulness. (B) Total sleep–wake amount during 12-hour dark (19:00–07:00 hours) and 12-hour light (07:00–19:00 hours) and the 24-hour period. (C–E) Sleep–wake architecture over 24 hours. Mean duration (C) and episode numbers (D) of NREM and REM sleep and wakefulness; transition numbers between S (NREM sleep), W (wakefulness), and R (REM sleep) stages (E). (F) Hourly slow-wave activity (SWA) of NREM sleep. *p < 0.05 versus control. Control (*n* = 7); lesion (*n* = 6). The horizontal filled and open bars on the x-axes indicate the 12-hour dark period and the 12-hour light period, respectively.
(TIF)

S3 Fig Expression of hM4Di receptors in the rostromedial tegmental nucleus (RMTg) mapped on coronal atlas drawings at four brain levels. The gray shading highlights the RMTg as shown in the Paxinos and Watson (2007) rat brain atlas. The expression of hM4Di receptors in the RMTg from each animal is outlined. *n* = 7 rats. IPN, interpeduncular nucleus; Pn, pontine nuclei.
(TIF)

S4 Fig The hM4Di-mediated inhibition of rostromedial tegmental nucleus (RMTg) neurons decreased NREM sleep in rats. (A) Coronal section shows the superimposed virus-injected area in seven rats numbered with the same color characters as the closed curves. The bilateral shaded areas indicate the rat RMTg locations. (B) A typical trace showed that Clozapine-N-oxide (CNO) application (horizontal bar) induced hyperpolarization and inhibited spontaneous firing of a neuron in the RMTg under whole-cell current-clamp. (C) Time course of NREM and REM sleep and wakefulness during the 12-hour light period (07:00–19:00 hours) in rats treated with saline or CNO at 09:00 hour. (D–H) During 1-hour post-injection period (9:00–10:00 hour) after saline or CNO injection in rats, episode numbers of each stage (D); number of NREM sleep bouts with different duration (E); transition numbers between S (NREM sleep), W (wakefulness), and R (REM sleep) stages (F); mean duration of each stage (G); slow-wave activity (SWA) of NREM sleep (H). *p < 0.05, **p < 0.01 versus saline by paired *t* test. CNO (0.3 mg/kg) was given by intraperitoneal (IP) injection at 09:00 h (*n* = 7). The horizontal open bar on the x axes indicate the 12-hour light period.
(TIF)

S5 Fig Activation of ventral tegmental area (VTA)/substantia nigra compacta (SNc) dopaminergic (DAergic) neurons decreased NREM sleep in tyrosine hydroxylase (TH)-Cre mice. (A, B) Drawings of superimposed AAV injection sites in the VTA (A, *n* = 8) and SNc (B, *n* = 8) of TH-Cre mice with different colors. (C) Clozapine-N-oxide (CNO) evoked vigorous firing of a VTA mCherry-positive neuron in a TH-Cre mouse. (D, E) c-Fos (green) was expressed in mCherry-positive (red) neurons after injection of 1 mg/kg CNO (D, E, top), but not injection of saline (D, E, bottom) in the VTA (D) and SNc (E). (F, G) Statistics of the co-expression of c-Fos and mCherry immunofluorescence of VTA (F) and SNc (G) DAergic neurons from TH-Cre mice (*n* = 3, per group). (H, J) Time course changes of NREM and REM sleep and wakefulness after intraperitoneal (IP) injection of saline or CNO (1.0 mg/kg) at 09:00 hour in hM3Dq-expressing TH-Cre mice in the VTA (H) and SNc (J). (I, K) Amount of each stage in 4 hours (I, VTA) or 3 hours (K, SNc) after saline or CNO injection. *p < 0.05, **p < 0.01 versus saline by paired *t* test. The horizontal open and filled bars on the x-axes indicate the 12-hour light and the 12-hour dark period, respectively.
(TIF)

S6 Fig Expression of hM3Dq receptors in the rostromedial tegmental nucleus (RMTg) mapped on coronal atlas drawings at four brain levels. The gray shading highlights the RMTg as shown in the Paxinos and Watson (2007) rat brain atlas. The expression of hM3Dq receptors in RMTg from each animal is outlined. *n* = 9 rats. IPN, interpeduncular nucleus; Pn, pontine nuclei.
(TIF)

## Abbreviations

AAV: adeno-associated virus
ACSF: artificial cerebrospinal fluid
ChR2: channelrhodopsin-2
CNO: clozapine-N-oxide
DA: dopamine
DREADD: designer receptor exclusively activated by designer drugs
DRN: dorsal raphe nucleus
EEG: electroencephalogram
GABA: γ-amino-butyric acid
Gscl: Goosecoid-like
IP: intraperitoneal
IPN: interpeduncular nucleus
IPSC: inhibitory postsynaptic current
LC: locus coeruleus
LDT: laterodorsal tegmental
M-R: muscarinic receptor
NREM: non-rapid eye movement
PPT: pedunculopontine tegmental
PTX: picrotoxin
REM: rapid eye movement
RMTg: rostromedial tegmental nucleus
SD: sleep deprivation
SNc: substantia nigra compacta
SWA: slow-wave activity
TH: tyrosine hydroxylase
VGAT: vesicular GABA transporter
VTA: ventral tegmental area
