# Supplementary figures and images for: The rostromedial tegmental nucleus is essential for non-rapid eye movement sleep
Source: PLoS Biol. 2018 Apr 13;16(4):e2002909. doi: 10.1371/journal.pbio.2002909 (PMC5919677; doi:10.1371/journal.pbio.2002909)

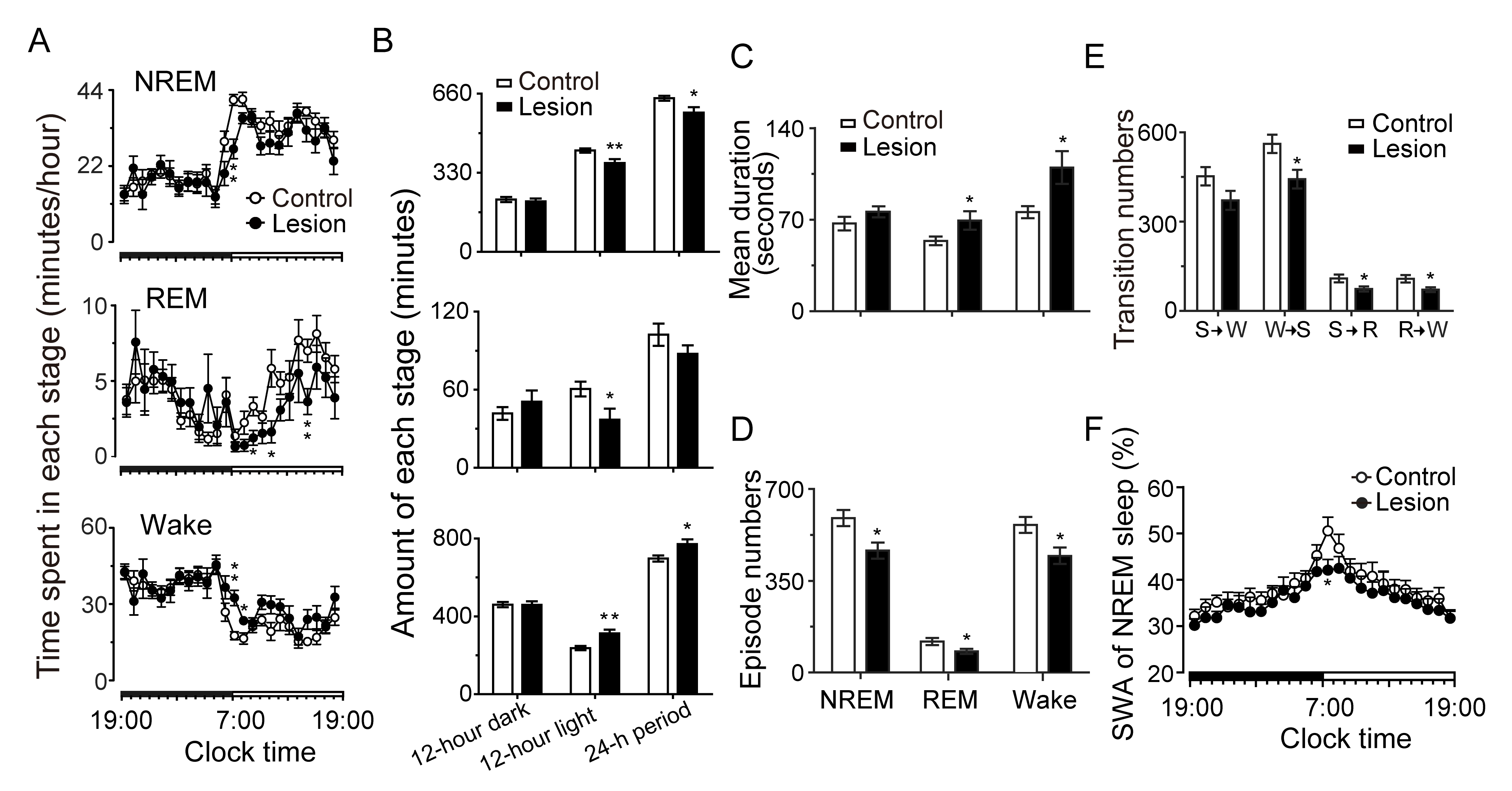

Supplement: S1 Fig — (A) Hourly amount of NREM and REM sleep and wakefulness. (B) Total sleep–wake amount during the 12-hour dark (19:00–7:00 hours) and the 12-hour light (07:00–19:00 hours) and the 24-hour period. (C–E) Sleep–wake architecture over 24 hours. Mean duration (C) and episode numbers (D) of NREM and REM sleep and wakefulness; transitions between S (NREM sleep), W (wakefulness), and R (REM sleep) stages (E). (F) Hourly slow-wave activity (SWA) of NREM sleep. *p < 0.05, **p < 0.01 versus control. Control (n = 10); lesion (n = 8). The horizontal filled and open bars on the x-axes indicate the 12-hour dark period and the 12-hour light period, respectively. (TIF) [file pbio.2002909.s002.tif]

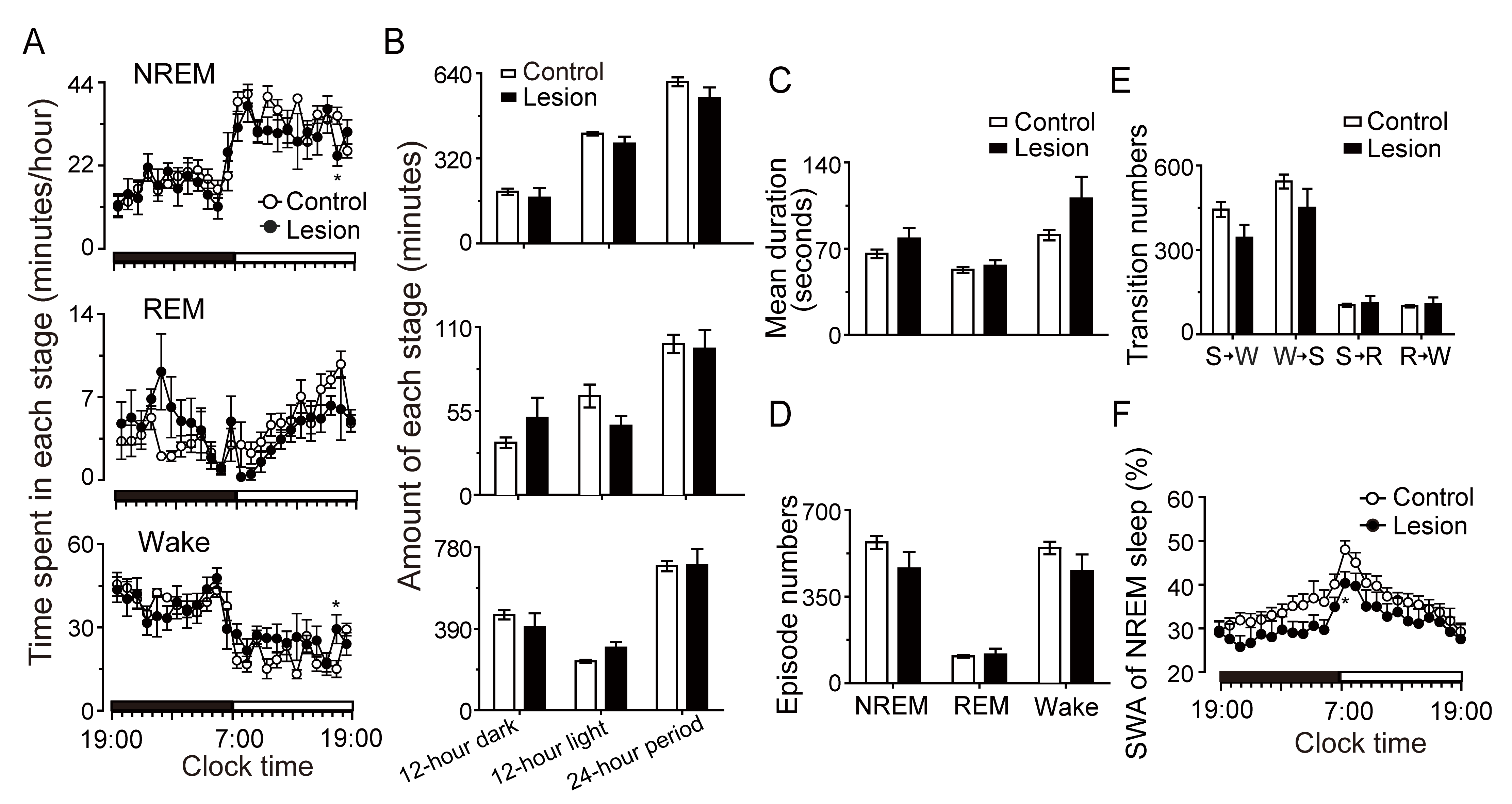

Supplement: S2 Fig — (A) Hourly amount of NREM and REM sleep and wakefulness. (B) Total sleep–wake amount during 12-hour dark (19:00–07:00 hours) and 12-hour light (07:00–19:00 hours) and the 24-hour period. (C–E) Sleep–wake architecture over 24 hours. Mean duration (C) and episode numbers (D) of NREM and REM sleep and wakefulness; transition numbers between S (NREM sleep), W (wakefulness), and R (REM sleep) stages (E). (F) Hourly slow-wave activity (SWA) of NREM sleep. *p < 0.05 versus control. Control (n = 7); lesion (n = 6). The horizontal filled and open bars on the x-axes indicate the 12-hour dark period and the 12-hour light period, respectively. (TIF) [file pbio.2002909.s003.tif]

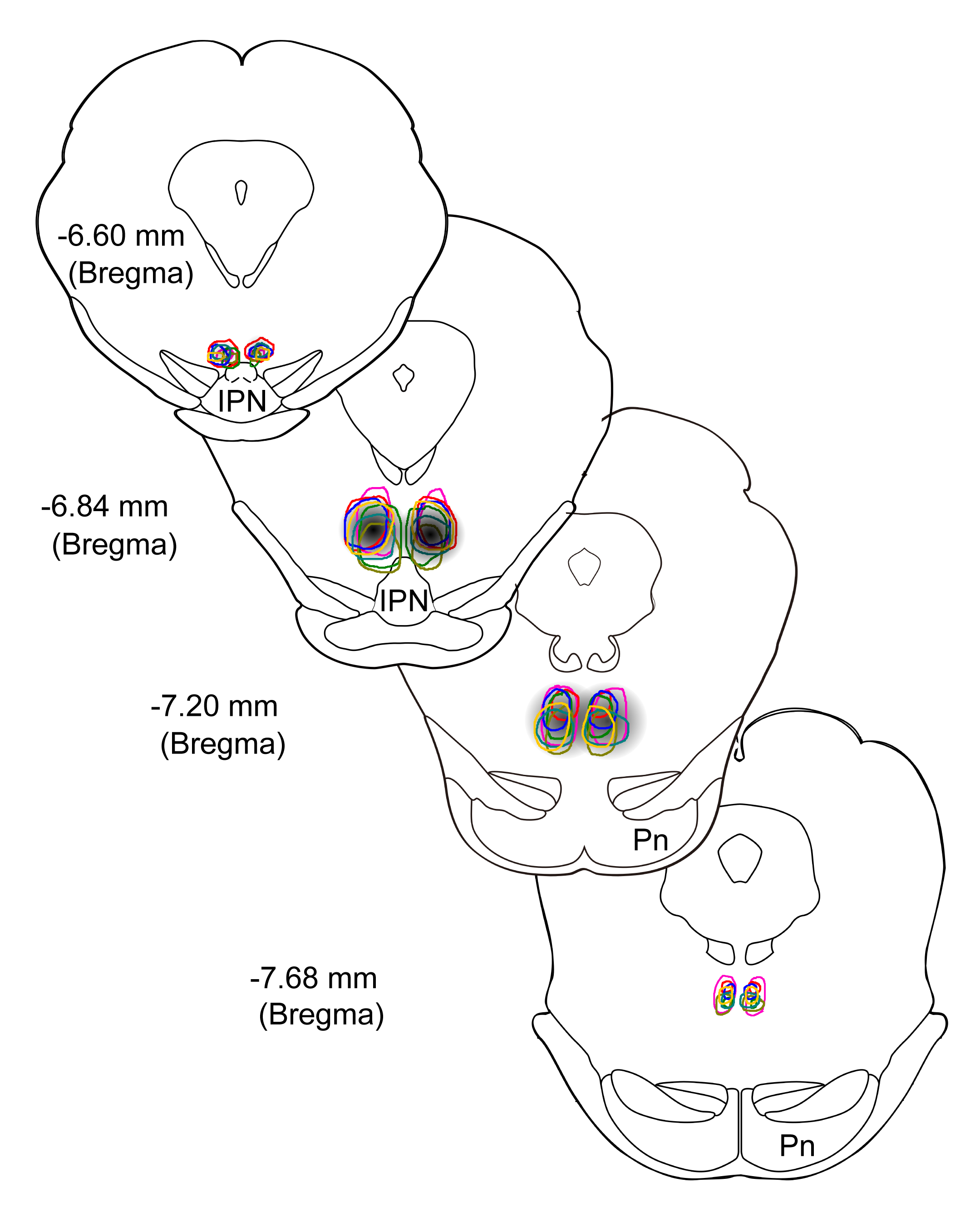

Supplement: S3 Fig — The gray shading highlights the RMTg as shown in the Paxinos and Watson (2007) rat brain atlas. The expression of hM4Di receptors in the RMTg from each animal is outlined. n = 7 rats. IPN, interpeduncular nucleus; Pn, pontine nuclei. (TIF) [file pbio.2002909.s004.tif]

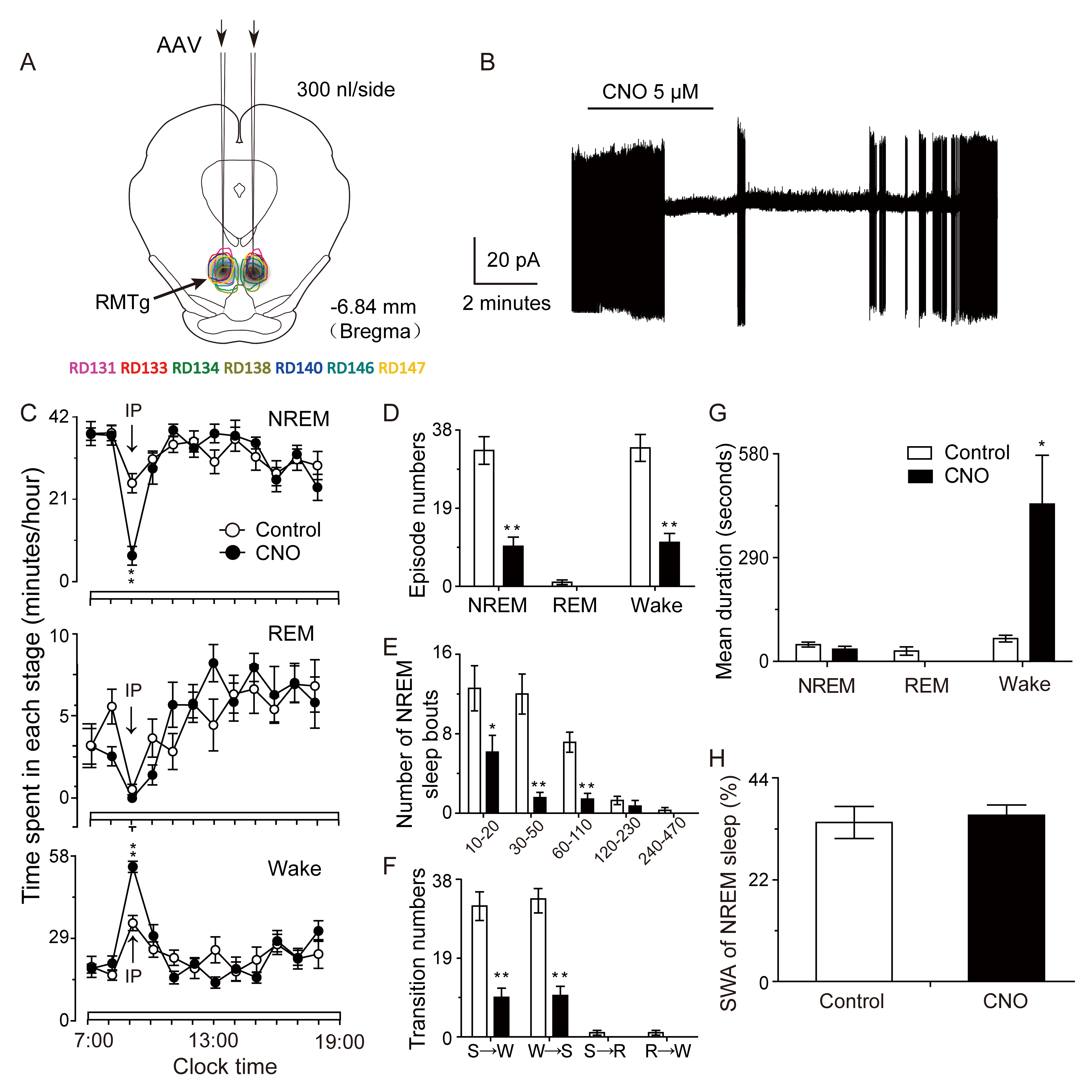

Supplement: S4 Fig — (A) Coronal section shows the superimposed virus-injected area in seven rats numbered with the same color characters as the closed curves. The bilateral shaded areas indicate the rat RMTg locations. (B) A typical trace showed that Clozapine-N-oxide (CNO) application (horizontal bar) induced hyperpolarization and inhibited spontaneous firing of a neuron in the RMTg under whole-cell current-clamp. (C) Time course of NREM and REM sleep and wakefulness during the 12-hour light period (07:00–19:00 hours) in rats treated with saline or CNO at 09:00 hour. (D–H) During 1-hour post-injection period (9:00–10:00 hour) after saline or CNO injection in rats, episode numbers of each stage (D); number of NREM sleep bouts with different duration (E); transition numbers between S (NREM sleep), W (wakefulness), and R (REM sleep) stages (F); mean duration of each stage (G); slow-wave activity (SWA) of NREM sleep (H). *p < 0.05, **p < 0.01 versus saline by paired t test. CNO (0.3 mg/kg) was given by intraperitoneal (IP) injection at 09:00 h (n = 7). The horizontal open bar on the x axes indicate the 12-hour light period. (TIF) [file pbio.2002909.s005.tif]

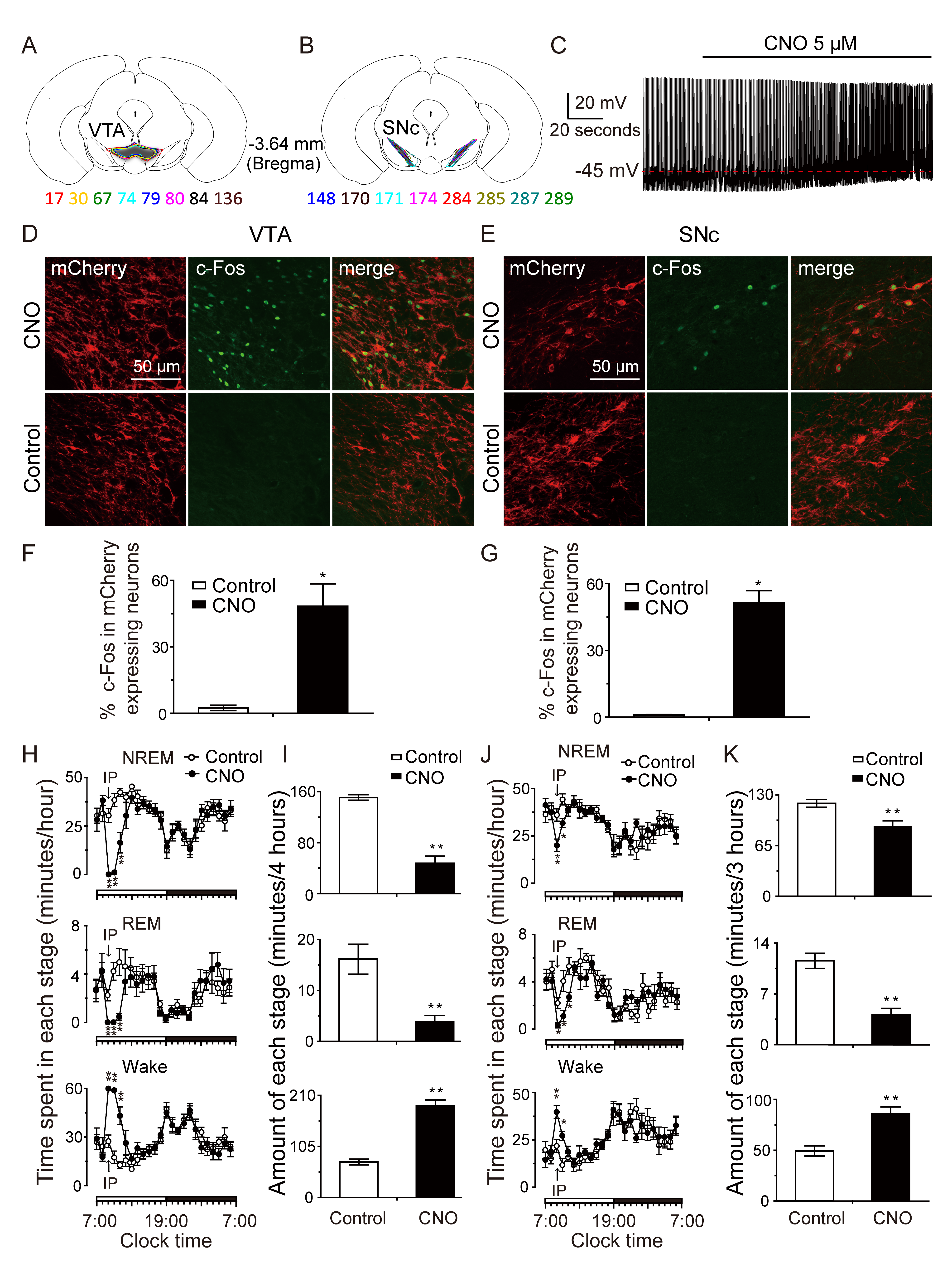

Supplement: S5 Fig — (A, B) Drawings of superimposed AAV injection sites in the VTA (A, n = 8) and SNc (B, n = 8) of TH-Cre mice with different colors. (C) Clozapine-N-oxide (CNO) evoked vigorous firing of a VTA mCherry-positive neuron in a TH-Cre mouse. (D, E) c-Fos (green) was expressed in mCherry-positive (red) neurons after injection of 1 mg/kg CNO (D, E, top), but not injection of saline (D, E, bottom) in the VTA (D) and SNc (E). (F, G) Statistics of the co-expression of c-Fos and mCherry immunofluorescence of VTA (F) and SNc (G) DAergic neurons from TH-Cre mice (n = 3, per group). (H, J) Time course changes of NREM and REM sleep and wakefulness after intraperitoneal (IP) injection of saline or CNO (1.0 mg/kg) at 09:00 hour in hM3Dq-expressing TH-Cre mice in the VTA (H) and SNc (J). (I, K) Amount of each stage in 4 hours (I, VTA) or 3 hours (K, SNc) after saline or CNO injection. *p < 0.05, **p < 0.01 versus saline by paired t test. The horizontal open and filled bars on the x-axes indicate the 12-hour light and the 12-hour dark period, respectively. (TIF) [file pbio.2002909.s006.tif]

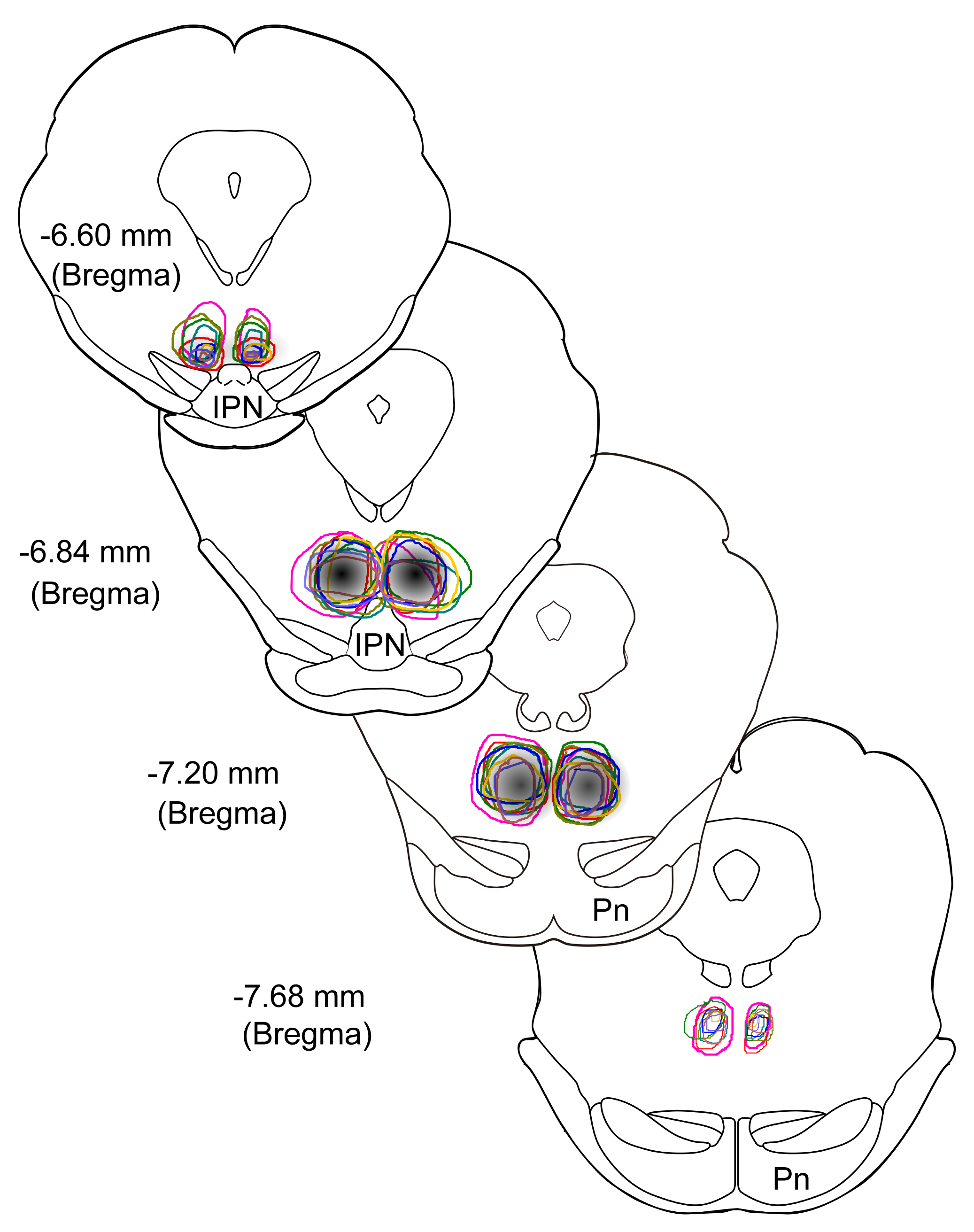

Supplement: S6 Fig — The gray shading highlights the RMTg as shown in the Paxinos and Watson (2007) rat brain atlas. The expression of hM3Dq receptors in RMTg from each animal is outlined. n = 9 rats. IPN, interpeduncular nucleus; Pn, pontine nuclei. (TIF) [file pbio.2002909.s007.tif]
